# Supplementary material for: Raman microspectroscopy and Raman imaging reveal biomarkers specific for thoracic aortic aneurysms
Source: Cell Rep Med. 2021 Apr 28;2(5):100261. doi: 10.1016/j.xcrm.2021.100261 (PMC8149374; doi:10.1016/j.xcrm.2021.100261)
Supplement: Document S1. Figures S1–S4 [file mmc1.pdf]

Cell Reports Medicine, Volume *2*

Supplemental information

Raman microspectroscopy and Raman imaging reveal biomarkers specific for thoracic aortic aneurysms

Kaori Sugiyama, Julia Marzi, Julia Alber, Eva M. Brauchle, Masahiro Ando, Yoshito Yamashiro, Bhama Ramkhelawon, Katja Schenke-Layland, and Hiromi Yanagisawa

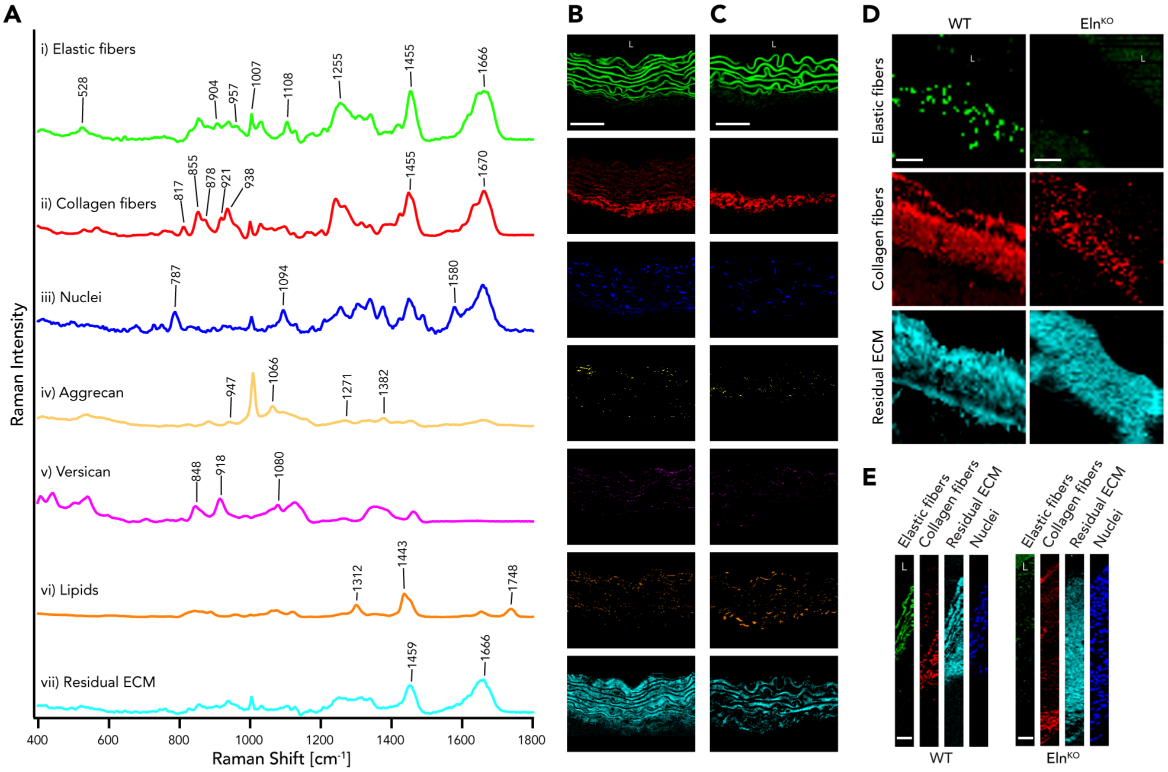

Supplemental Figure S1. **Raman imaging of cross sections of murine ascending and descending aortas by true component analysis (TCA), and confirmation of the specificity of the elastic fiber reference spectrum. Related to Figure 1.** (A) Identified spectral components correlate to elastic fibers (green), collagen fibers (red), nuclei (blue), aggrecan (yellow), versican (pink), lipids (orange), and residual ECM (cyan). (B) Corresponding intensity distribution heatmaps for each component of ascending aorta. Scale bars equal 50 µm. (C) Corresponding intensity distribution heatmaps of descending aorta. Luminal (L) side to the top. Scale bars equal 50 µm. (D) Large area and (E) high-resolution Raman scans of WT and *Eln*^{KO} at postnatal day 1 and TCA false-color intensity distribution images for elastic fibers (green), collagens (red), ECM (cyan) and nuclei (blue). Scale bars equal 40 µm for large area scans. Scale bars equal 25 µm for high resolution scans. (See also Supplemental Table S1)

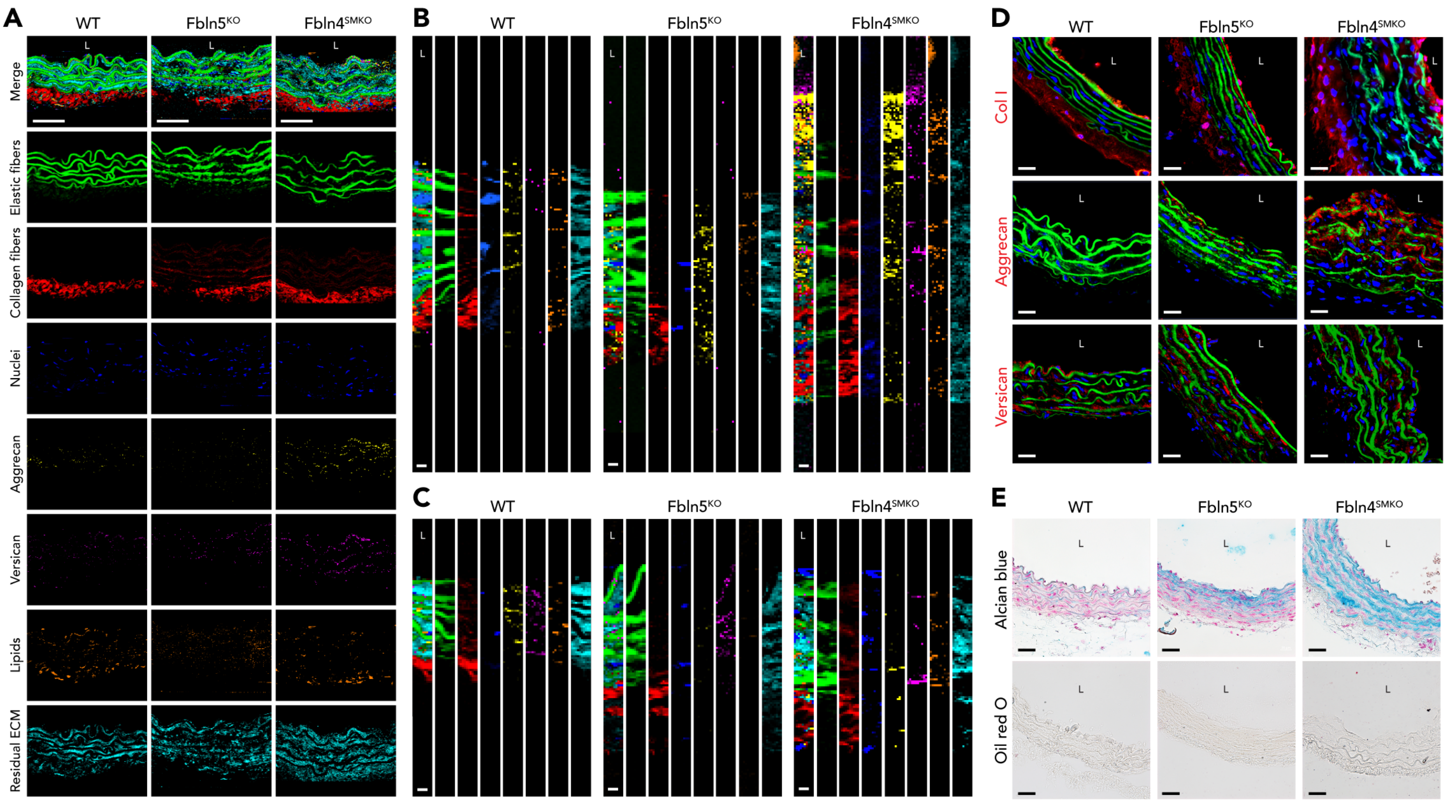

Supplemental Figure S2. Representative large area and high-resolution Raman images and immunofluorescence (IF) staining and histochemistry of WT, *Fbln5^KO^*, and *Fbln4^SMKO^* descending aortas. Related to Figure 1. (A) TCA false-color intensity distribution heatmaps for elastic fibers (green), collagen fibers (red), nuclei (blue), aggrecan (yellow), versican (pink), lipids (orange) and residual ECM (cyan) in adult WT, *Fbln5^KO^*, and *Fbln4^SMKO^* descending aortas. Scale bars equal 50 µm. (B-C) Raman images of high-resolution scans in (B) ascending and (C) descending aortic tissues for elastic fibers (green), collagen fibers (red), nuclei (blue), aggrecan (yellow), versican (pink), lipids (orange) and residual ECM (cyan). Scale bars equal 5 µm. (D) IF staining for collagen type Ⅰ, aggrecan, and versican (red), elastin autofluorescence (green) and nuclei (blue). Scale bars equal 20 µm. (E) Routine histochemical staining for Alcian blue (glycosaminoglycans) and Oil red O (lipid). Scale bars equal 20 µm.

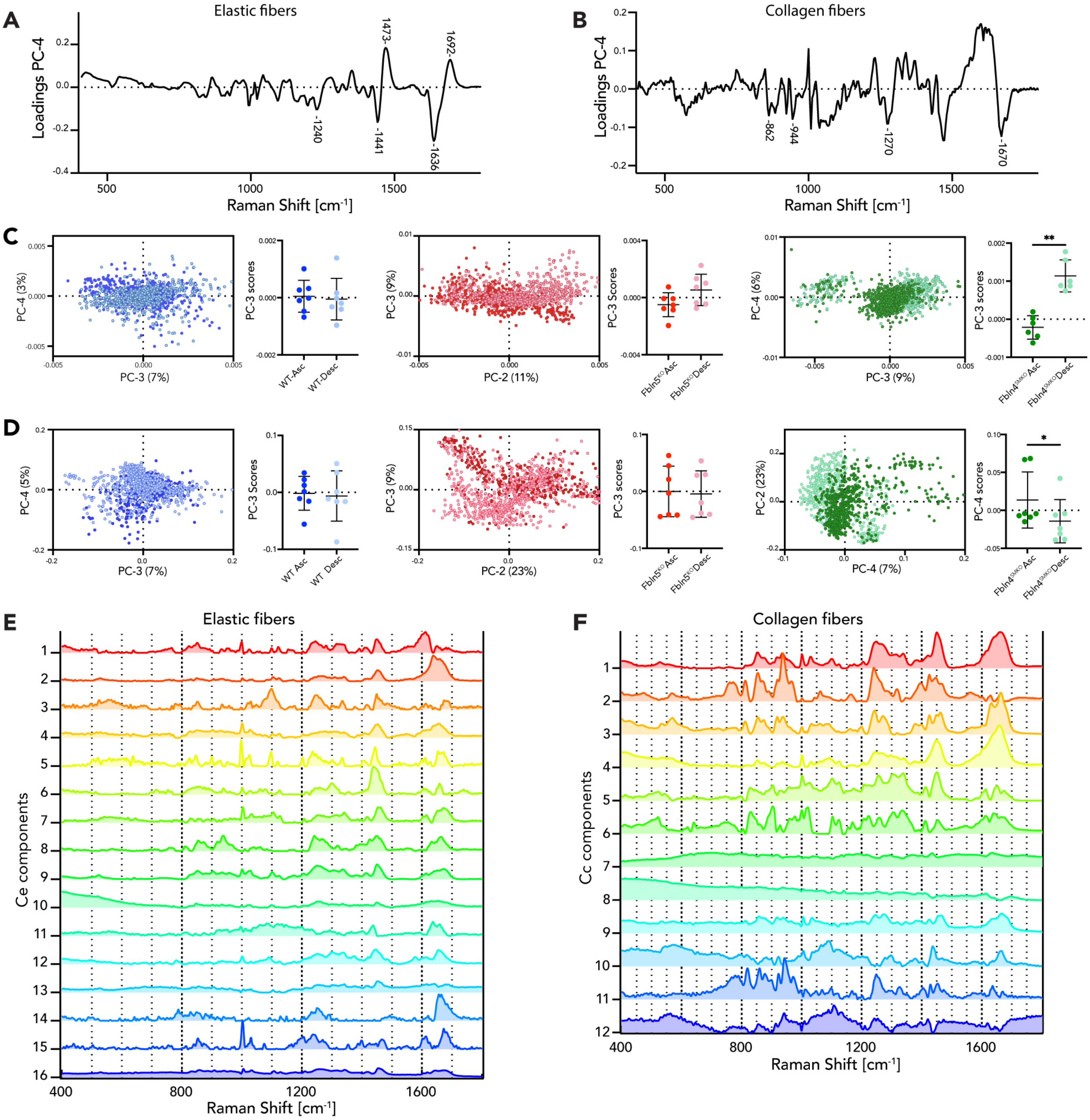

Supplemental Figure S3. Murine MVA by PCA and MCR. Related to Figures 2, 3, and 4. (A-B) PC loadings plots of (A) elastic fibers and (B) collagen fibers in murine ascending aortas shown in Figure 2. (C-D) PCA score plots of murine (C) elastic fibers and (D) collagen fibers comparing ascending (Asc) and descending (Desc) aortas in WT, *Fbln5*^{KO}, and *Fbln4*^{SMKO}. Both, elastic fibers and collagen fibers showed the separation between ascending and descending aortas in *Fbln4*^{SMKO}. Statistical analysis was performed by paired t-test (elastic fibers in WT, *Fbln5*^{KO}, and *Fbln4*^{SMKO}, and collagen fibers in WT and *Fbln5*^{KO}) and Wilcoxon test (collagen fibers in *Fbln4*^{SMKO}). *$p<0.05$, **$p<0.01$. N ≥ 6 per genotype. (E-F) MCR decomposed components of (E) elastic fibers (Ce1-Ce16 components) and (F) collagen fibers (Cc1-Cc12 components) of WT, *Fbln5*^{KO}, and *Fbln4*^{SMKO}. (See also Supplemental Table S2)

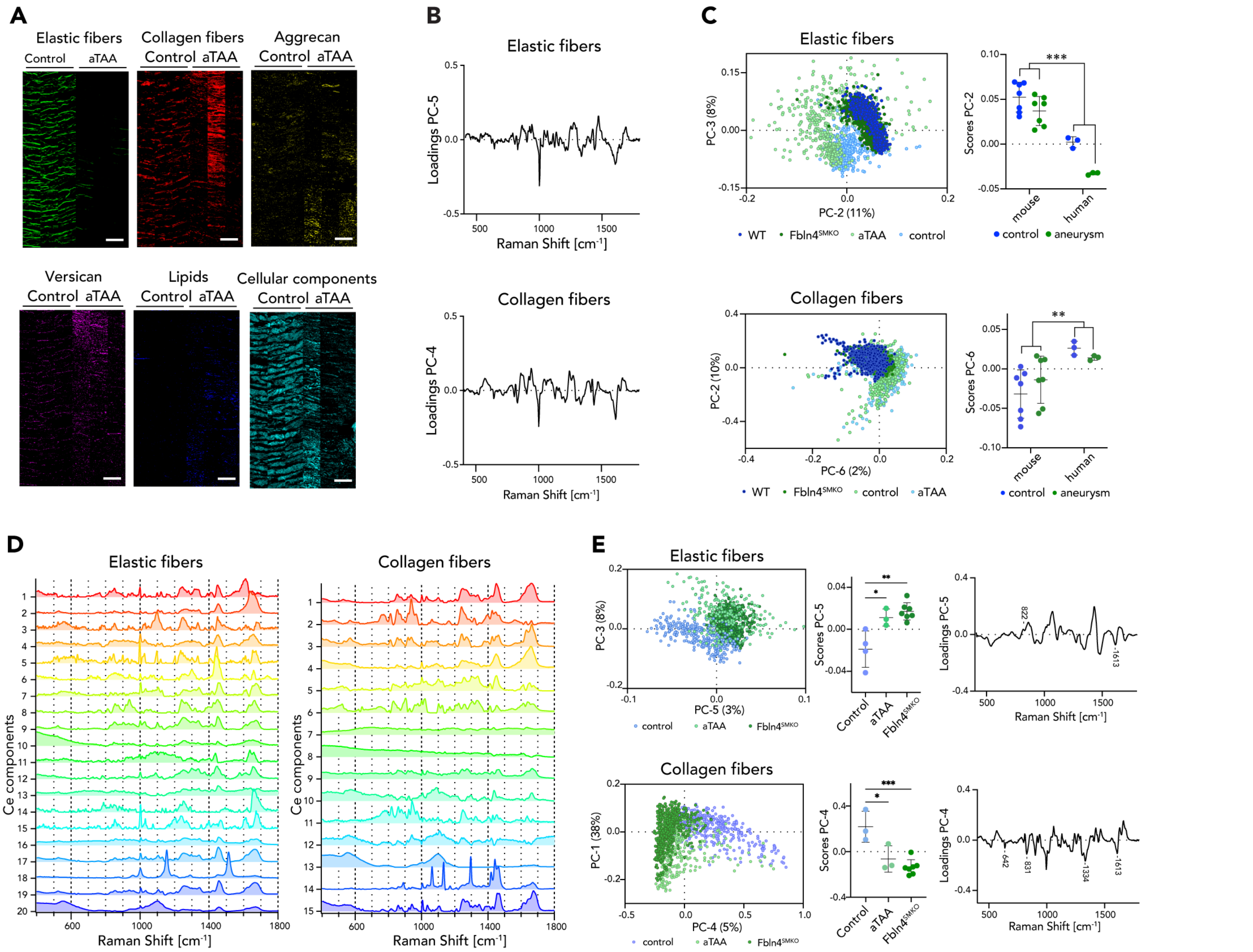

Supplemental Figure 4. Human and murine MVA by TCA, PCA, and MCR for control and aTAA aortas. Related to Figures 5 and 6. (A) TCA Raman imaging of human control aortas and aTAA obtained by murine reference spectra. Scale bars equal 50 µm. (B) Corresponding loadings plots to PCAs of human data (Figures 5B and 6B, respectively) performed in elastic fibers and collagen fibers. (C) PCA of murine and human tissues reveal species-specific differences in elastic fibers and collagens. PC-3 vs PC-2 scores plots of murine and human aortic elastic fibers. Statistical analysis of mean score values. PC-2 vs PC-6 scores plots of murine and human aortic collagen fibers. Statistical analysis of mean score values. N ≥ 6 (murine) and n ≥ 3 (human), data represent mean score values ± SD, two-way ANOVA, **p<0.01, ***p<0.001. (D) Human MCR decomposed spectra from elastic fibers (Ce1-Ce20) and collagen fibers (Cc1-Cc15). (E) PCA identified clustering of principal components of human and murine aneurysm structures from human control. (upper panel) Elastic fiber PCA of control human, aTAA, and $Fbln4^{SMKO}$ for PC-3 vs PC-5 scores plots, statistical analysis of PC-5 (mean score values ± SD), and loadings plots of PC-5. (lower panel) Collagen fiber PCA comparing control human, aTAA, and $Fbln4^{SMKO}$ scores plots, statistical analysis of PC-4 (mean score values ± SD), and loadings plots of PC-4. N ≥3, one-way ANOVA, *; p<0.05, **p<0.01, ***p<0.001. (See also Supplemental Table S2)
