# Supplementary material for: Raman microspectroscopy and Raman imaging reveal biomarkers specific for thoracic aortic aneurysms
Source: Cell Rep Med. 2021 Apr 28;2(5):100261. doi: 10.1016/j.xcrm.2021.100261 (PMC8149374; doi:10.1016/j.xcrm.2021.100261)
Supplement: Document S2. Article plus supplemental information [file mmc4.pdf]

Cell Reports
Medicine

Article

# Raman microspectroscopy and Raman imaging reveal biomarkers specific for thoracic aortic aneurysms

## Graphical abstract

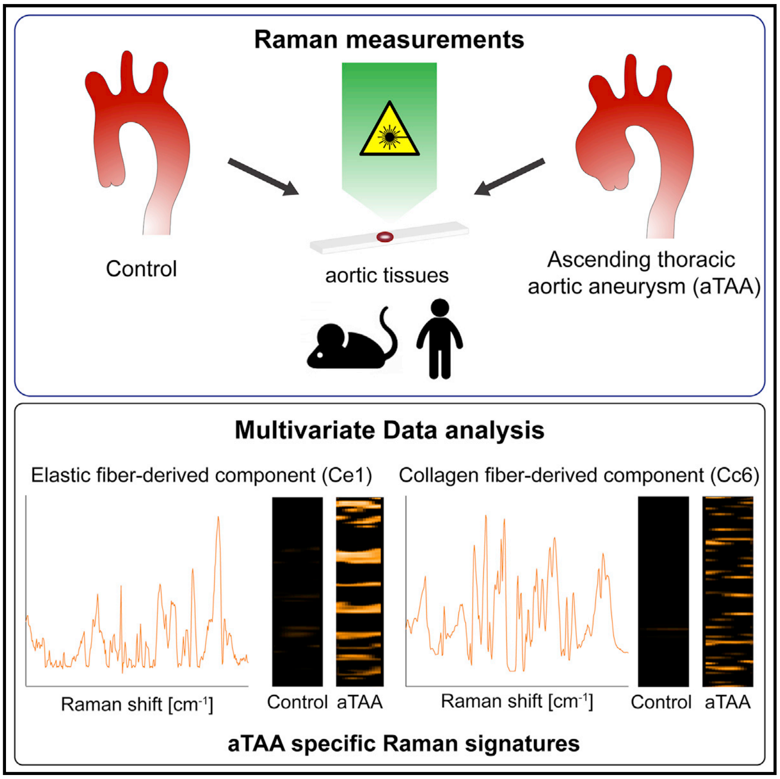


## Authors

Kaori Sugiyama, Julia Marzi, Julia Alber, ..., Bhama Ramkhelawon, Katja Schenke-Layland, Hiromi Yanagisawa

## Correspondence

katja.schenke-layland@uni-tuebingen.de (K.S.-L.), hkyanagisawa@tara.tsukuba.ac.jp (H.Y.)

## In brief

Sugiyama et al. performed label-free Raman imaging of human and murine ascending thoracic aortic aneurysms (aTAAs). Using Raman spectra for multivariate data analysis, they identify spectral biomarkers for aTAA in elastic fibers and collagen fibers, Ce1, and Cc6, respectively, that are significantly increased in aTAA lesions.

## Highlights

- Label-free Raman imaging of human/murine ascending thoracic aortic aneurysm (aTAA)
- Multivariate analysis of Raman spectra allows detection of aTAA molecular features
- Identification of spectral biomarkers for aTAA in elastic and collagen fibers
- Alterations in amino acid spectra correlate with aTAA formation

Sugiyama et al., 2021, Cell Reports Medicine 2, 100261
May 18, 2021 © 2021 The Author(s).
https://doi.org/10.1016/j.xcrm.2021.100261

Cell Reports Medicine

CellPress
OPEN ACCESS

Article

# Raman microspectroscopy and Raman imaging reveal biomarkers specific for thoracic aortic aneurysms

Kaori Sugiyama,[1,2,11] Julia Marzi,[3,4,5,11] Julia Alber,[3] Eva M. Brauchle,[3,4,5] Masahiro Ando,[6,7] Yoshito Yamashiro,[1] Bhama Ramkhelawon,[8] Katja Schenke-Layland,[3,4,5,9,*] and Hiromi Yanagisawa[1,10,12,*]

[1]Life Science Center for Survival Dynamics, Tsukuba Advanced Research Alliance (TARA), University of Tsukuba, Tsukuba, Japan
[2]Institute for Advanced Research of Biosystem Dynamics, Research Institute for Science and Engineering, Waseda University, Tokyo, Japan
[3]Department of Women's Health, Research Institute of Women's Health, Eberhard Karls University, Tübingen, Germany
[4]NMI Natural and Medical Sciences Institute at the University of Tübingen, Reutlingen, Germany
[5]Cluster of Excellence iFIT (EXC 2180) "Image-Guided and Functionally Instructed Tumor Therapies," University of Tübingen, Tübingen, Germany
[6]Research Organization for Nano & Life Innovation, Waseda University, Tokyo, Japan
[7]Japan Science and Technology Agency, PRESTO, Saitama, Japan
[8]Division of Vascular Surgery, Department of Surgery and Department of Cell Biology, New York University Langone Health, New York, NY, USA
[9]Department of Medicine/Cardiology, Cardiovascular Research Laboratories, David Geffen School of Medicine at University of California, Los Angeles, Los Angeles, CA, USA
[10]Department of Basic Medical Science, Faculty of Medicine, University of Tsukuba, Tsukuba, Ibaraki, Japan
[11]These authors contributed equally
[12]Lead contact
*Correspondence: katja.schenke-layland@uni-tuebingen.de (K.S.-L.), hkyanagisawa@tara.tsukuba.ac.jp (H.Y.)
https://doi.org/10.1016/j.xcrm.2021.100261

## SUMMARY

Aortic rupture and dissection are life-threatening complications of ascending thoracic aortic aneurysms (aTAAs), and risk assessment has been largely based on the monitoring of lumen size enlargement. Temporal changes in the extracellular matrix (ECM), which has a critical impact on aortic remodeling, are not routinely evaluated, and cardiovascular biomarkers do not exist to predict aTAA formation. Here, Raman microspectroscopy and Raman imaging are used to identify spectral biomarkers specific for aTAAs in mice and humans by multivariate data analysis (MVA). Multivariate curve resolution-alternating least-squares (MCR-ALS) combined with Lasso regression reveals elastic fiber-derived (Ce1) and collagen fiber-derived (Cc6) components that are significantly increased in aTAA lesions of murine and human aortic tissues. In particular, Cc6 detects changes in amino acid residues, including phenylalanine, tyrosine, tryptophan, cysteine, aspartate, and glutamate. Ce1 and Cc6 may serve as diagnostic Raman biomarkers that detect alterations of amino acids derived from aneurysm lesions.

## INTRODUCTION

Ascending thoracic aortic aneurysms (aTAAs) are life threatening because of sudden rupture and dissection without any detectable symptoms.[1] The aortic diameter in aTAA increases at least 50% compared to the normal thoracic aorta,[2] and the maximum aortic diameter is used as a criterion for surgical repair. However, patients with an aortic diameter of <5.5 cm could develop acute dissection,[3] indicating an unmet need for diagnostic tools that consider the integrity of the aneurysmal wall.

General risk factors of aortic aneurysms are smoking, hypertension, inflammation, hyperlipidemia, and aging. aTAAs are frequently associated with mutations in components of the extracellular matrix (ECM), including fibrillin-1 in Marfan syndrome,[4] type III $\alpha$1-collagen in Ehlers-Danlos type III,[5] lysyl hydroxylase 1 in Ehlers-Danlos type XI,[6] and fibulin-4 (FBLN4 encoded by *EFEMP2*) in cutis laxa syndrome.[7,8] Activation of the transforming growth factor $\beta$ (TGF-$\beta$) pathway is observed in Marfan syndrome and Loeys-Dietz syndrome.[9] In addition, mutations in the genes involved in the generation of contractile forces in smooth muscle cells (SMCs) have been identified in familial TAAs and dissections.[10] Therefore, the causal genes for TAAs encompass the ECM to cellular components along the elastin-contractile units.[11]

Medial necrosis and disruption of elastic fibers are characteristic pathological features of human aTAA. Massive accumulations of proteoglycans, such as aggrecan and versican,[12] and phenotypic switching of SMCs have been observed,[13] which can be caused by alterations in ECM components, including FBLN4,[14] fibulin-5,[15] and collagen VIII.[16] These observations suggest that pathological changes involved in aneurysms


© 2021 The Author(s).
This is an open access article under the CC BY license (http://creativecommons.org/licenses/by/4.0/).

include cellular and extracellular changes in the vessel wall in addition to increased lumen size.

FBLN4 and fibulin-5 (FBLN5) are major elastic fiber-associated proteins and are essential for elastogenesis *in vivo*.[17] Mutations of *FBLN4* and *FBLN5* have been identified in autosomal recessive cutis laxa type I patients, and *FBLN4* was shown to be associated with aTAA.[18] Previously, we generated SMC-specific *Fbln4* knockout mice (*Fbln4$^{SMKO}$*),[14] which develop aortic aneurysms that recapitulate the human condition. We also generated *Fbln5* KO mice (*Fbln5$^{KO}$*), which develop aortic elongation and tortuosity but do not form aortic aneurysms.[19] Lack of *Fbln4* resulted in abnormal collagen maturation in addition to abnormal elastic fibers in the *Fbln4$^{SMKO}$* aorta,[20] which was attributed to decreased lysyl oxidase-mediated cross-linking of collagen fibrils.[21,22]

Raman microspectroscopy and Raman imaging are marker-independent and non-destructive imaging methods evolving in the biological and biomedical fields.[23,24] Raman microspectroscopy can distinguish different components in tissues by detecting molecular vibrations. ECM molecules such as elastin, collagens, or proteoglycans have specific Raman spectra.[25,26] In previous studies, Raman microspectroscopy was shown to reveal differences between healthy and cancerous tissues in the skin[27,28] and lungs,[29] and age-dependent differences in the ECM surrounding pancreatic islets.[30] Moreover, Raman microspectroscopy and Raman imaging have the potential to analyze cardiovascular ECM structures on a molecular level.[31–33] At this time, no reports are available regarding the assessment of aortic aneurysm by Raman measurements.

In this study, we used marker-independent Raman microspectroscopy and Raman imaging combined with multivariate data analysis (MVA), including true component analysis (TCA), principal-component analysis (PCA), and multivariate curve resolution (MCR), to examine structural and molecular signatures with a high spatial resolution among *Fbln4$^{SMKO}$*, *Fbln5$^{KO}$*, and wild-type (WT) aortic tissues. Furthermore, we extended our analyses to human aTAA based on the spectral dataset obtained from murine aneurysmal tissues.

## RESULTS

### Raman imaging allows marker-independent detection of aortic structural composition

To set the reference spectra of aortic tissues, we performed Raman imaging on cross-sections of murine WT ascending aortas at 1 month of age. We successfully extracted spectra by TCA and five major spectral components were identified, which were assigned to the Raman signatures of elastic fibers, collagen fibers, nuclei, lipids, and residual ECM (Figure S1A, i, ii, iii, vi, and vii).[25,34] In addition, reference spectra of lyophilized aggrecan and versican were added as defined components to TCA (Figure S1A, iv and v). In elastic fibers, relevant Raman bands were detected at 528, 957, and 1,108 cm$^{-1}$ for desmosine and isodesmosine; 904 cm$^{-1}$ for C-C-N stretch; 1,255 cm$^{-1}$ for amide III; 1,455 cm$^{-1}$ for $CH_3$ and $CH_2$ deformation; and 1,666 cm$^{-1}$ for amide I.[25] In collagen fibers, relevant Raman bands were found at 817 cm$^{-1}$ for C-C stretch[35]; 855, 878, 921, and 938 cm$^{-1}$ for proline and hydroxyproline[36]; and 1,670 cm$^{-1}$ for amide I.[25] In nuclei, nucleotides and phosphate backbone-related Raman bands were detected at 787 cm$^{-1}$, 1,094 cm$^{-1}$, and 1,580 cm$^{-1}$.[34] In aggrecan, relevant Raman bands were present at 947 cm$^{-1}$ for C-C deformation,[37] 1,066 cm$^{-1}$ for C-C and C-O stretch,[38] 1,271 cm$^{-1}$ for amide III,[39] and 1,382 cm$^{-1}$ for $CH_2$ deformation.[37] In versican, 848 cm$^{-1}$ correlated with glycosaminoglycans,[40] 918 cm$^{-1}$ to glycogen,[41] and 1,080 cm$^{-1}$ to $SO_3^-$ symmetric stretch.[37] In lipids, relevant Raman bands were 1,312 cm$^{-1}$ for $CH_3CH_2$ twisting mode,[42,43] 1,443 cm$^{-1}$ for $CH_2$ deformation,[43] and 1,748 cm$^{-1}$ for C=O stretch.[43] The residual ECM component was dominated by protein Raman bands found at 1,459 and 1,666 cm$^{-1}$ correlating with $CH_2$ and $CH_3$ deformation and amide I, respectively.[36,44] These identified spectra were used as reference spectra for subsequent TCAs (Table S1). Intensity distribution heatmaps for each component were generated for Raman maps of ascending (Figure S1B) and descending (Figure S1C) aortas.

To confirm the sensitivity and specificity of the identified Raman signature for elastic fibers, aortic tissues of postnatal day (P) 1 WT and *Eln$^{KO}$*[45] pups were analyzed. Previously identified core reference spectra were used to perform TCA on the large area and high-resolution scans on cross-sections of WT and *Eln$^{KO}$* aortas (Figures S1D and S1E). Analysis of WT aortas resulted in Raman images that were positive for the applied reference spectra of elastic fibers, whereas signals were undetectable in *Eln$^{KO}$* aortas, indicating that the identified spectra were highly specific to elastic fibers.

### Transfer of WT spectral components to Fbln5$^{KO}$ and Fbln4$^{SMKO}$ aortic tissues

We used two established murine models of aortic disease, *Fbln5$^{KO}$* and *Fbln4$^{SMKO}$*, to examine whether Raman imaging can detect an alteration of ECM in the diseased aortic wall.[14,19] Spectral components identified in the WT aorta were applied as reference spectra to generate Raman images of the mutant aortas and to define the distribution and orientation of each component in ascending (Figure 1A) and descending (Figure S2A) aortas. In Raman imaging, WT aortas contained solid native elastic fibers, whereas *Fbln5$^{KO}$* elastic fibers showed disruptions. *Fbln4$^{SMKO}$* elastic fibers were disorganized, and elastic layers were markedly increased in the ascending aortas. In WT and *Fbln5$^{KO}$*, collagen fibers were predominantly located in the adventitia, whereas collagen fibers were expanded from adventitia to medial layers in *Fbln4$^{SMKO}$* aorta. WT, *Fbln5$^{KO}$*, and *Fbln4$^{SMKO}$* showed a similar distribution of nuclei. Aggrecan, which was previously shown to be increased in TAA patients,[12] was markedly accumulated in *Fbln4$^{SMKO}$* aortas. In contrast, distribution of versican was similar among genotypes. Lipid signals were marginally increased in the *Fbln4$^{SMKO}$* aortas compared with WT. The imaging results obtained from descending aortas were comparable to ascending aortas, except for *Fbln4$^{SMKO}$*, which showed a less destructed organization of elastic fibers and a WT-like organization of collagen fibers when compared with ascending aortas (Figure S2A). High-resolution scans were also used to examine the structural details (Figures S2B and S2C) as well as for in-depth spectral analysis by PCA and

CellPress
OPEN ACCESS

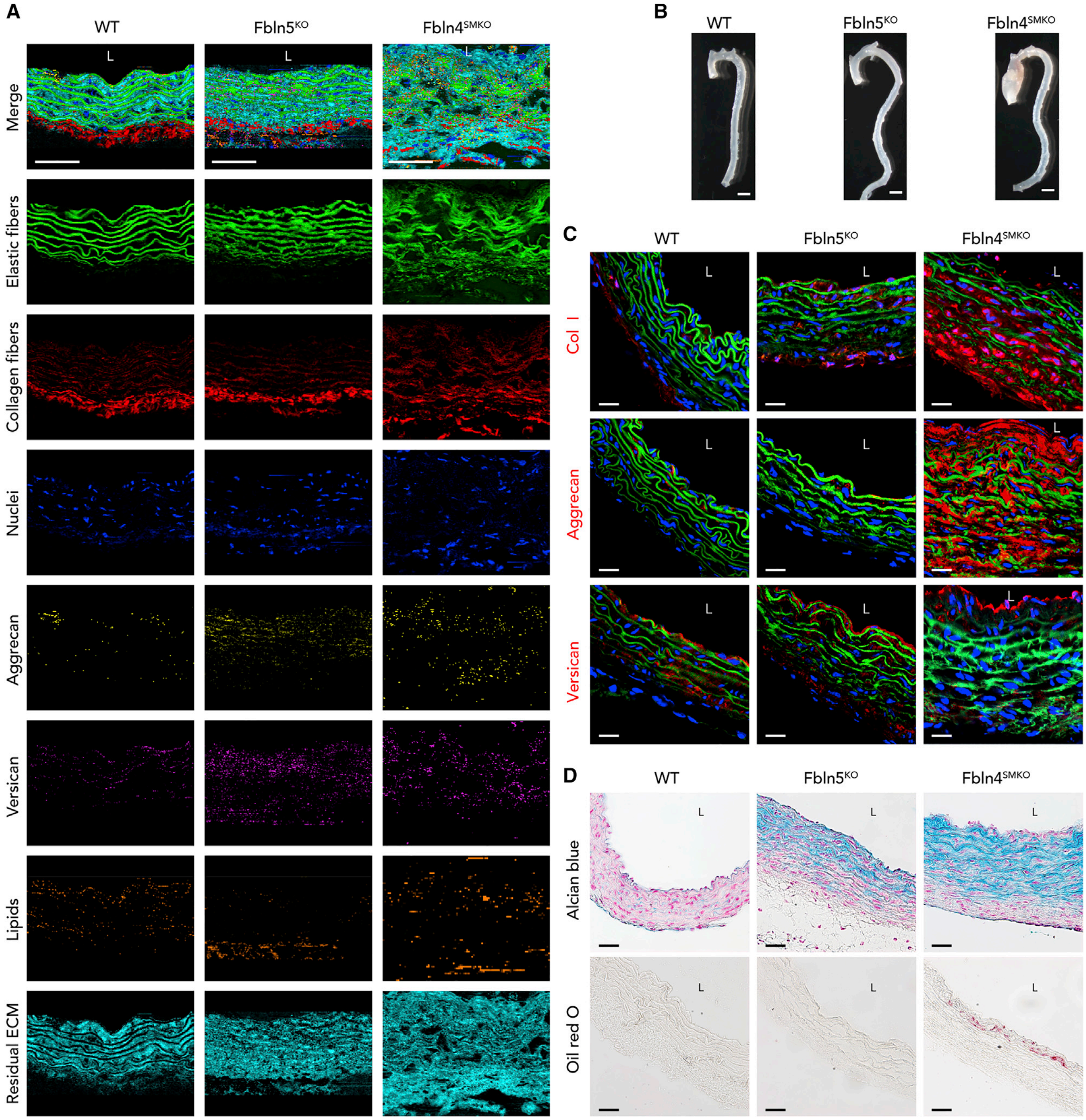


**Figure 1. Representative Raman images and immunofluorescence (IF) staining and histochemistry for WT, fibulin-5 knockout (*Fbln5*$^{KO}$), and smooth muscle-specific fibulin-4 knockout (*Fbln4*$^{SMKO}$) mice**

(A) Raman images of large-area scans. Cross-sections of the ascending aorta from WT, *Fbln5*$^{KO}$, and *Fbln4*$^{SMKO}$. False-color intensity distribution heatmaps for elastic fibers (green), collagen fibers (red), nuclei (blue), aggrecan (yellow), versican (pink), lipids (orange), and residual ECM (cyan). Scale bars, 50 μm. see also Figure S1.

(B) Gross photos of murine aortas of WT, *Fbln5*$^{KO}$, and *Fbln4*$^{SMKO}$ mice. *Fbln5*$^{KO}$ exhibits elongation of the ascending aorta and tortuous descending aorta. *Fbln4*$^{SMKO}$ shows a large aneurysm in the ascending aorta but not in the descending aorta. Scale bars, 1 mm.

(C) IF staining for collagen type I, aggrecan, and versican in red, elastin autofluorescence (green), and nuclei (blue). Scale bars, 20 μm.

(D) Routine histochemical staining for Alcian blue (glycosaminoglycans) and oil red O (lipid) staining.

Scale bars, 20 μm. L, luminal side.

MCR analysis. Gross observation of *Fbln5*$^{KO}$ and *Fbln4*$^{SMKO}$ showed elongation and tortuosity of the aorta in both animals, whereas aneurysm was seen only in the ascending aorta of *Fbln4*$^{SMKO}$, as was previously reported (Figure 1B).[14,19]

## Routine histology complies with Raman imaging results

Immunofluorescence (IF) staining and histological analyses were performed to compare and evaluate the performance of Raman imaging for the identification of tissue structures as well as distribution of matrix components in the aortic tissues. IF identified collagen type I (Col I), aggrecan, versican, elastic fibers (autofluorescence), and nuclei (DAPI) in ascending aortic tissues (Figure 1C). Whereas Col I localized in the adventitia of the WT aorta, its distribution expanded into medial layers of *Fbln5*$^{KO}$, and this change was exacerbated in *Fbln4*$^{SMKO}$, exhibiting a strong and broad distribution throughout the aortic wall. For aggrecan staining, *Fbln4*$^{SMKO}$ aorta showed strong signals in the intima as well as in the medial layers, and versican staining was positive in the intima and adventitia of all genotypes.

Next, histochemical staining was performed using Alcian blue and oil red O (ORO), which visualizes glycosaminoglycans and lipids, respectively (Figure 1D). Alcian blue staining exhibited a massive accumulation of proteoglycans in *Fbln5*$^{KO}$ and *Fbln4*$^{SMKO}$, but not in WT. ORO staining showed small lipid deposition in the *Fbln4*$^{SMKO}$ aortas, but not in other genotypes. Histology and IF staining of the descending aortas were similar to those of the ascending aortas (Figures S2D and S2E). The structures and distribution patterns identified by Raman imaging correlated with routine immunohistochemical staining.

## PCA discriminated the molecular composition of the aortic tissues among WT, Fbln5$^{KO}$, and Fbln4$^{SMKO}$

To access spectral patterns in WT, *Fbln5*$^{KO}$, and *Fbln4*$^{SMKO}$, we focused on elastic fibers. We used the Raman spectra extracted from the high-resolution scanning Raman images of elastic fibers in ascending aortas for PCA to determine the grouping of the datasets (Figure 2A). The scores plot of PC-4 against PC-5 showed a clustering of all genotypes (Figure 2B). Statistical analysis of the PC-4 score indicated a significant difference in WT versus *Fbln4*$^{SMKO}$ and *Fbln5*$^{KO}$ versus *Fbln4*$^{SMKO}$ (Figure 2C; Table S2). The loadings plot, which represents Raman shift values of the selected PC, was further analyzed for the corresponding spectral information that was responsible for the clustering of elastic fibers (Figure S3A) and collagen fibers (Figure S3B). The PC-4 loadings plot represented Raman features in elastic fibers dominating in WT (negative loadings bands) or *Fbln4*$^{SMKO}$ (positive loadings), respectively (Figure S3A). Those features referred to a decrease in the amide III group (1,240 cm$^{-1}$) and spectral shifts in the $CH_2$ (1,441–1,473 cm$^{-1}$) and amide 1 (1,640–1,690 cm$^{-1}$) bands upon elastic fiber deterioration. PCA on single spectra extracted from the collagen component in ascending aorta Raman images of high-resolution scans showed separation in PC-4 versus PC-3 scores plot (Figures 2D and 2E). PC-4 demonstrated a significant difference between WT and *Fbln4*$^{SMKO}$ as well as *Fbln5*$^{KO}$ (Figure 2F). The corresponding molecular differences identified in the PC-4 loadings plot showed collagen-related peaks for proline and hydroxyproline (862 and 944 cm$^{-1}$) and amide III (1,270 cm$^{-1}$) and amide III (1,670 cm$^{-1}$) bands (Figure S3B).

Additional PCAs and statistical analyses were performed to compare elastic fiber and collagen composition between ascending and descending aortas in WT, *Fbln5*$^{KO}$, and *Fbln4*$^{SMKO}$ (Figures S3C and S3D; Table S2). While WT and *Fbln5*$^{KO}$ showed no differences within the regions, *Fbln4*$^{SMKO}$ showed significant separation between ascending and descending aorta for both elastic fibers (Figure S3C) and collagen fibers (Figure S3D).

## Decomposition of elastic and collagen fibers by MCR

Since PCA clearly differentiated elastic and collagen fibers in WT, *Fbln5*$^{KO}$, and *Fbln4*$^{SMKO}$, we used MCR to further determine the submolecular structures specific to aneurysm phenotype by Raman microspectroscopy. First, Raman spectra focusing on elastic fibers were decomposed into 16 components (Ce1–Ce16) by MCR and corresponding Raman images were generated (Figure S3E). Statistical analysis of the relative intensity of MCR Raman images revealed that Ce1 was significantly different in *Fbln4*$^{SMKO}$ compared with WT and *Fbln5*$^{KO}$ (Figure 3; Table S2). As Figure 3 and Table S2 show, Ce1 and Ce9 contained Raman bands arising from proteins, such as phenylalanine (1,002 cm$^{-1}$), amide III (1,246–1,257 cm$^{-1}$), amide I (1,662–1,672 cm$^{-1}$), and $CH_2/CH_3$ deformation (1,450–1,454 cm$^{-1}$).[34] Ce1 is assigned to a non-elastin-related substructure of the elastic fiber because of a lack of desmosine and isodesmosine bands. Ce9 demonstrated a similar Raman spectrum to the native elastic fiber component identified by TCA (Figure S1A) and protein-related bands (Table S1).[25,34]

We then analyzed collagen fibers for differences in molecular signatures among genotypes. MCR extracted TCA-derived collagen fibers to 12 components (Cc1–Cc12) in ascending aortas of WT, *Fbln5*$^{KO}$, and *Fbln4*$^{SMKO}$ (Figure S3F). Cc1 was comparable to collagen fibers indicated by proline and hydroxyproline bands (Figure 4A; Table S2).[25,36] Statistical analysis of the intensity of each MCR image showed significant differences in Cc6 (Table S2). Cc6 was only detectable in *Fbln4*$^{SMKO}$, indicating the abnormal molecular modification specific to *Fbln4*$^{SMKO}$ (Figures 4B and 4C). This suggests that the different structure of collagen fibers is related to aortic aneurysm phenotype.

Based on the differential MCR analysis, we further searched for aneurysm-specific and region-specific molecular signatures focusing on collagen fibers in WT and *Fbln4*$^{SMKO}$ (Figure 4D). Statistical analysis (Table S2) revealed significant differences between ascending and descending aortas of WT and *Fbln4*$^{SMKO}$ in Cc6, indicating that these bands detected the aneurysm-specific changes in *Fbln4*$^{SMKO}$ aortas (Figures 4D and 4E). In addition, Cc6 was only detected in the ascending aortas of *Fbln4*$^{SMKO}$, and the overall spectral pattern of Cc6 includes protein-indicating bands such as $CH_2/CH_3$ deformation and amide I/III (Table S1). The Cc6 spectrum contains amino acid residues, including phenylalanine at 616, 1,003, 1,222, and 1,613 cm$^{-1}$; tyrosine at 642, 833, 1,170, and 1,222 cm$^{-1}$; tryptophan at 749, 1,018, 1,336, and 1,455 cm$^{-1}$; cysteine at 515 cm$^{-1}$; and aspartic and glutamic acid at 1,412 cm$^{-1}$ (tentative assignments).[34,46] Interestingly, Cc6 showed relatively intense peaks

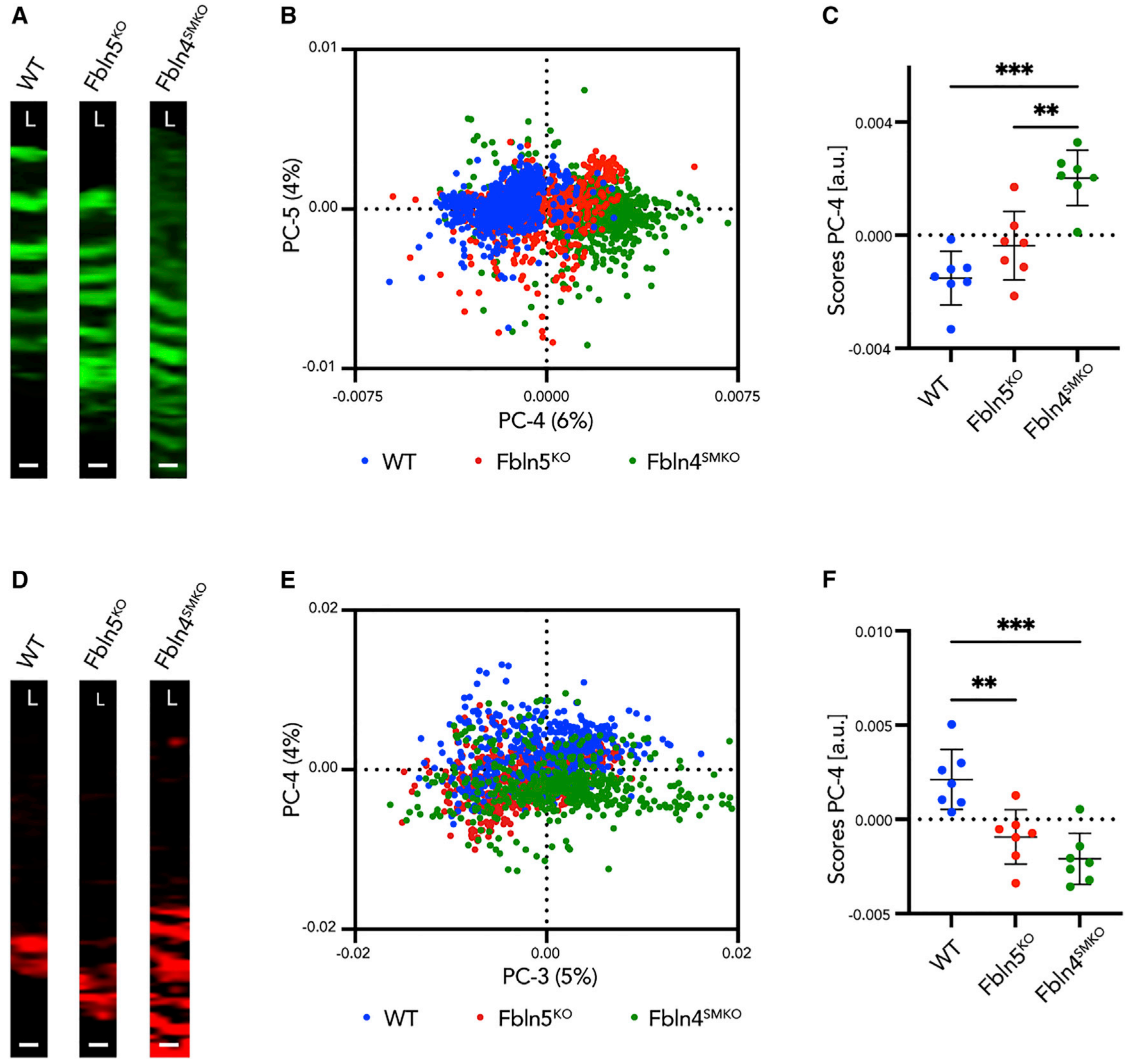


**Figure 2. Principal-component analysis (PCA) on elastic fibers and collagen fibers**

(A) Region of interest (ROI) for single spectra extraction defined by elastic fiber TCA component of high-resolution scans in WT, *Fbln5 KO*, and *Fbln4*$^{SMKO}$.

(B) PCA scores plot of PC-4 versus PC-5 shows a clustering between WT (blue), *Fbln5*$^{KO}$ (red), and *Fbln4*$^{SMKO}$ (green).

(C) Statistical analysis of PC-4 scores indicated as mean score values ± SD.

(D) ROI for single spectra extraction defined by collagen fiber TCA component of high-resolution scans in WT, *Fbln5 KO*, and *Fbln4*$^{SMKO}$.

(E) PCA scores plot of PC-3 versus PC-4 shows a clustering between WT (blue), *Fbln5*$^{KO}$ (red), and *Fbln4*$^{SMKO}$ (green).

(F) Statistical analysis of PC-4 scores indicated as mean score values ± SD.

Scale bars, 5 μm. L, luminal side. N ≥ 6 animals per genotype. Multiple comparison 1-way ANOVA; $^{**}p \leq 0.01$, $^{***}p \leq 0.001$.

See also Figure S3A and S3B and Table S2.

of tryptophan, suggesting dysregulated tryptophan metabolisms or a substitution of tryptophan in Col I, which has been observed in abdominal aortic aneurysm (AAA).[47,48]

## Identification of disease-related MCR Raman signature in human aTAA

To further examine whether the aTAA-relevant Raman signature in murine aneurysms is applicable to human aTAAs, we analyzed tissues from aTAA patients. TCA reference components defined in the murine WT tissues described in Figure S1A were applied for TCA and subsequent Raman image generation of human control and aTAA tissues (Figure S4A). Spectra of elastic fibers and collagen fibers were then extracted based on the TCA intensity distribution heatmaps. The PCA results showed a separation between control and aTAA in both elastic fibers (Figures 5A– 5C) and collagen fibers (Figures 6A–6C, S4B, and S4C).

For MCR analysis, the murine MCR components from elastic fibers (Ce1–Ce16) were used as reference spectra and additional spectral components, which were only detected in human tissues (Ce17–Ce20; Figure S4D), were extracted. As Table S2 shows, Ce1 in aTAA samples is significantly different from

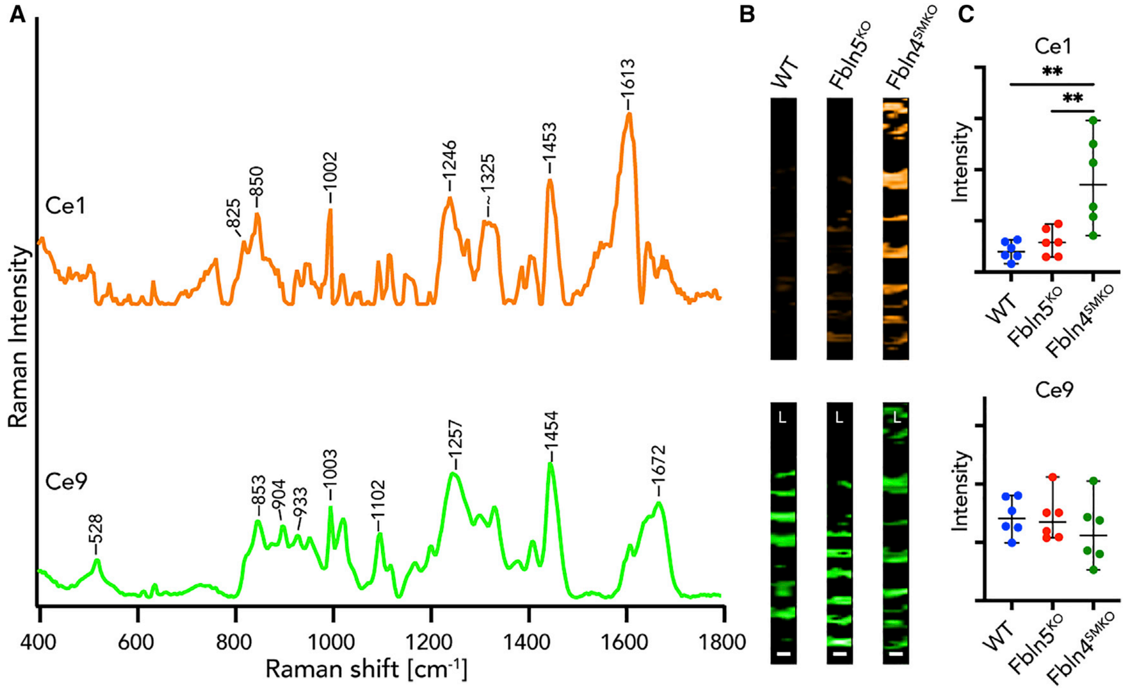


**Figure 3. Multivariate curve resolution (MCR) decomposed spectra and images of elastic fibers in WT, *Fbln5$^{KO}$*, and *Fbln4$^{SMKO}$***

(A) Most relevant MCR spectra in *Fbln4$^{SMKO}$* compared with WT and *Fbln5$^{KO}$*. Ce1 is specifically found in *Fbln4$^{SMKO}$* (orange) and Ce9 represents the standard elastic fiber signature (green).

(B) MCR images of Ce1 and Ce9 components.

(C) Statistical analysis of relative intensities of MCR images in Ce1 and Ce9.

Scale bars, 5 μm. L, luminal side. N = 6 per genotype, data points are represented as mean intensity values ± SD, statistical analysis by 1-way ANOVA; **$p < 0.01$. See also Figure S3E and Tables S1 and S2.

control samples. In particular, Ce1 was increased in both murine and human aneurysms (Figures 5D and 5E).

Similarly, we examined aneurysm lesion-specific spectra in collagen fibers (Figure 6). The murine collagen MCR components (Cc1–12) were used as reference spectra for human analysis. Similar to the elastic fiber analysis, additional components were extracted only from the human data (Cc13–15; Figure S4D). Significant differences were revealed in Cc6 between control and aTAA (Table S2). Cc6 was increased in human aneurysms similar to murine aneurysms (Figure 6E).

In addition, we used PCA to compare changes in elastic fibers and collagen fibers in murine aneurysm and human aTAA. Although significant species differences were detected (Figure S4C), aneurysm tissues from mice and humans were separated from healthy human controls (Figure S4E). A significant separation of control human aortas and aTAA, as well as *Fbln4$^{SMKO}$*, was shown in PC-5 for elastic fibers and in PC-4 for collagen fibers. Moreover, the corresponding loadings recapitulated the aneurysm-specific peaks identified by MCR. Elastin loadings demonstrated peaks at ~822 and 1,613 cm$^{-1}$ similar to Ce1 (Figure 5D). Collagen loadings demonstrated peaks at 642, 831, 1,334 and 1,613 cm$^{-1}$ characteristic for bands identified in MCR component Cc6 (Figure 6D). MCR further decomposed elastic fiber and collagen fiber components and identified aneurysm-specific molecular signatures in human aTAA, which have the potential for use as diagnostic markers.

## DISCUSSION

In this study, we used Raman microspectroscopy and Raman imaging to perform marker-independent analyses of ascending aortic aneurysms in a murine model and human aTAA. Raman imaging allowed the visualization of cellular and extracellular components with a high spatial resolution. Structural features of aortic tissues in their native conditions were identified without the need for using specific labeling. The obtained data correlated to the distributional patterns demonstrated by IF staining and histochemistry, and sample preparation was simple and less time-consuming compared with conventional staining procedures. MVA further allowed for submolecular discrimination between healthy aortas and aneurysmal lesions, and successfully identified human aneurysm-specific marker signatures in elastic fibers (Ce1) and collagen fibers (Cc6) that can be used as biomarkers for aTAA diagnosis.

Raman microspectroscopy has the ability to evaluate tissues *in situ* in their native conditions.[49] For ECM molecules, which are usually incorporated in a complex tissue surrounded by assembled and complex fibrous structures, Raman microspectroscopy may facilitate examination in a more native-like state. Marker-independent biomedical methods, such as echocardiography and X-ray microtomography, are a gold standard in clinical settings, but are limited to image-based analysis. Raman measurements are advantageous among optical methods

CellPress
OPEN ACCESS

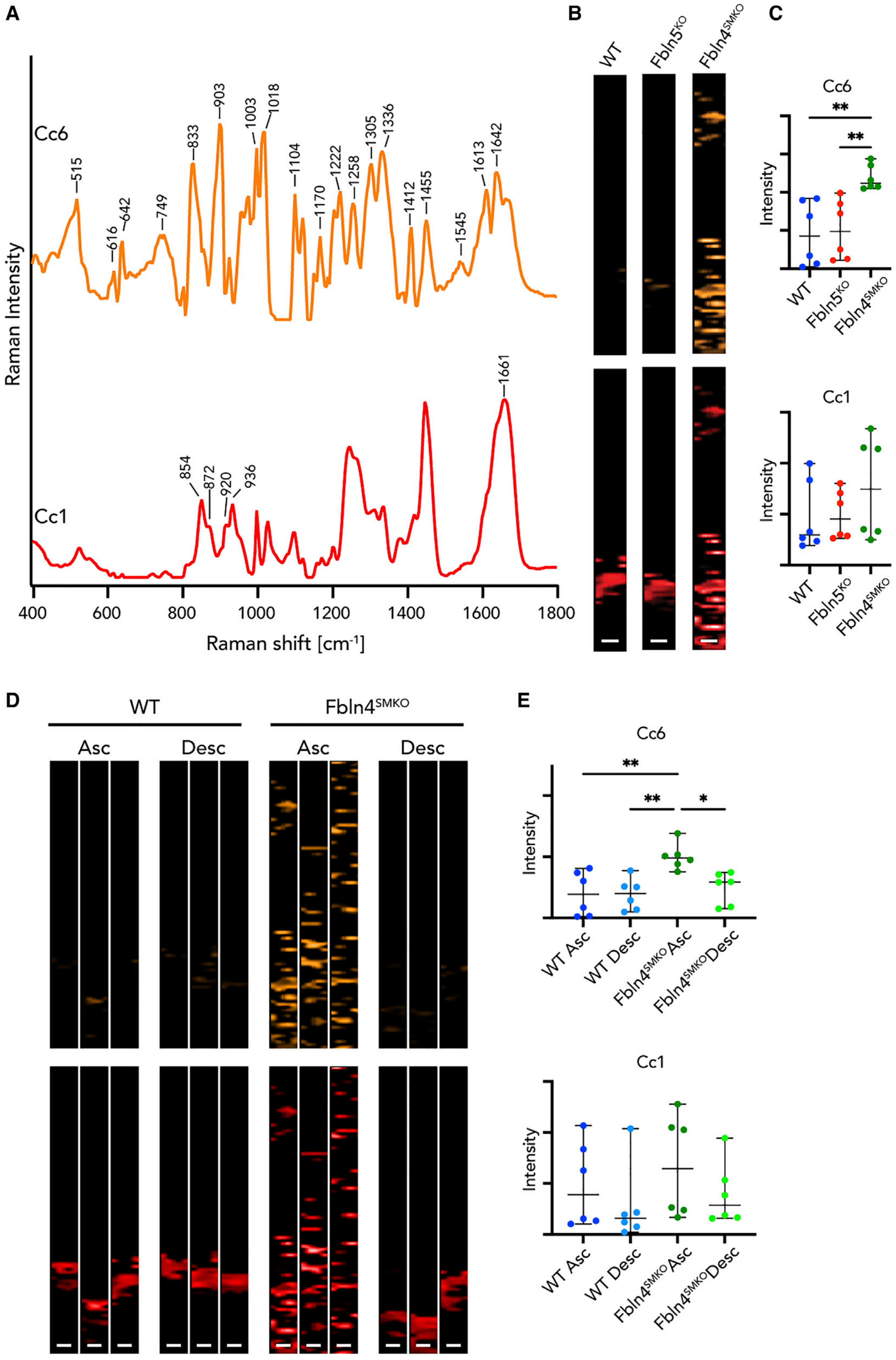


(legend on next page)

because they provide hyperspectral images with additional information on molecular structures. By combining massive spectral datasets with deep learning techniques, the accuracy of disease-specific signatures and the definition of tissue borders is increasing.

### Structural differences of aortic tissues revealed by TCA and PCA

Elastic fibers in *Fbln5*$^{KO}$ and *Fbln4*$^{SMKO}$ appeared similarly disrupted at a light microscopic level; however, Raman imaging and PCA separated the genotypes at PC-4 (Figure 2C). Likewise, PCA separated ascending aortas from descending aortas in *Fbln4*$^{SMKO}$ (Figures S3C and S3D). The molecular analysis of elastic fibers by PCA identified differences in Raman bands of amide I and amide III between *Fbln5*$^{KO}$ and *Fbln4*$^{SMKO}$. Shifts in amide I and III have been previously reported in relation to an increase in α-helical content in elastic fibers due to structural deterioration.[50] Since electron microscopic observation failed to distinguish the morphological differences in elastic fibers between ascending and descending aortas in *Fbln4*$^{SMKO}$ (data not shown), this suggests that Raman microspectroscopy can be a powerful tool to study the alteration of elastic fiber components from the view of secondary structural alteration.[51]

For collagen fibers, differences in distributional patterns identified by IF staining in WT, *Fbln5*$^{KO}$, and *Fbln4*$^{SMKO}$ were also identified by marker-independent spectral scans. Raman imaging combined with PCA not only provides semiquantitative information on vascular collagen fibers but also allows the simultaneous assessment of qualitative molecular-level differences. PCA revealed a separation of WT, *Fbln5*$^{KO}$, and *Fbln4*$^{SMKO}$, and PC loadings showed different structures of proteins in *Fbln5*$^{KO}$ and *Fbln4*$^{SMKO}$, which are possibly due to differences in proline and hydroxyproline content in collagen fibers.[25,36] As for elastic fibers, alterations in the features of collagen fibers were apparent between aneurysmal ascending aortas and non-aneurysmal descending aortas in *Fbln4*$^{SMKO}$. In addition to providing information regarding molecule localization and quantity, which can also be accessed by routine staining, Raman measurements allow for the identification of minor alterations in ECM structure, indicating the potential as an early diagnostic tool to identify pre-aneurysmal lesions in the aortic wall.

In normal aortas, proteoglycans such as versican are distributed transmurally and contribute to the regulation of residual stress.[52] Alcian blue staining demonstrated the increased deposition of glycosaminoglycans (GAGs) in *Fbln5*$^{KO}$ and *Fbln4*$^{SMKO}$, suggesting that disrupted elastic fibers may facilitate the deposition of GAGs or that accumulation of GAG affects the structural integrity of elastic fibers. Aggrecan accumulation was massive in *Fbln4*$^{SMKO}$ compared with *Fbln5*$^{KO}$ and WT. Aggrecan is specifically expressed in vascular SMCs and is involved in vascular remodeling.[53] Since the deposition of GAGs causes the distortion of mechanical stress and can create a spot for aortic dissection,[54] early detection of GAG accumulation and pathogenic proteoglycan within the aortic wall may be a potential diagnostic tool for diseased aorta at risk of dissection.

### Identification of aneurysm-specific spectra by MCR

MCR effectively decomposed TCA elastic fibers and collagen fibers and identified aneurysm-specific Raman signals. In elastic fibers, Ce1 appears to be derived from abnormal proteins associated with aneurysmal lesions, which is detectable only in aTAA and *Fbln4*$^{SMKO}$. Although the comparison of Raman spectra and accurate assignment to authentic proteins are needed, the 1,613 $cm^{-1}$ band in Ce1 could be a characteristic marker for aneurysm, since the band does not overlap with amide I or phenylalanine.

Although elastic fibers have been suggested as a major site of pathological alteration in *Fbln4*$^{SMKO}$ aortas,[14] MCR identified the region-specific features of aTAA for murine and human aortas not only in elastic structures but also in collagen fibers. Previously, we reported that different types of collagens showed distinctive peaks related to $CH_2$ deformation at 1,453 and 1,456 $cm^{-1}$.[30] Intriguingly, Cc6, a protein-like component, was detected only in murine and human aTAA regions (Figures 4 and 6). The spectrum of Cc6 features high intensities of several amino acid residues, indicating compositional changes of collagen molecules. Amino acid profiles are shown to be associated with aortic aneurysm and dissection in human patients.[48] MCR has successfully captured distinct marker spectra related to aTAA, enabling the specification of structural and compositional changes in elastin and collagen molecules.

### Raman measurement as a diagnostic tool for aTAA

In the present study, PCA revealed distinct Raman signatures between ascending and descending aortas in *Fbln4*$^{SMKO}$ based on the molecular structure of ECM components, highlighting the sensitivity of Raman microspectroscopy for the detection of lesion-specific biochemical differences that are not accessible by other routine techniques. In addition, two-step decompositions in TCA and MCR were efficient ways to analyze the details and proper features of spectral components. Although there were differences in species and tissue conditioning such as paraffin embedding and cryosection, TCA was robust enough to localize ECM structures with a single set of reference

**Figure 4. MCR decomposed spectra and images of collagen fibers in ascending (Asc) and descending (Desc) aortas of WT, *Fbln5*$^{KO}$, and *Fbln4*$^{SMKO}$**

(A) Most relevant MCR spectra in *Fbln4*$^{SMKO}$ compared with WT and *Fbln5*$^{KO}$. Cc6 was identified as a specific Raman spectrum in *Fbln4*$^{SMKO}$ (orange), and Cc1 represents the native signature of collagen fibers (red).

(B) MCR images of Cc6 and Cc1 components.

(C) Statistical analysis of relative intensities of MCR images in Cc6 and Cc1.

(D) MCR images of Cc6 and Cc1 in WT and *Fbln4*$^{SMK}$ in ascending and descending aortas. Three representative images per genotype.

(E) Statistical analysis of relative intensities of MCR images in Cc6 and Cc1.

Scale bars, 5 μm. L, luminal side. N = 6 animals per genotype, data points represent mean intensity values of 3 images ± SD, 1-way ANOVA (Cc6) or Kruskal-Wallis test (Cc1) were performed; $^*p < 0.05$, $^{**}p < 0.01$.

See also Figure S3F and Tables S1 and S2.

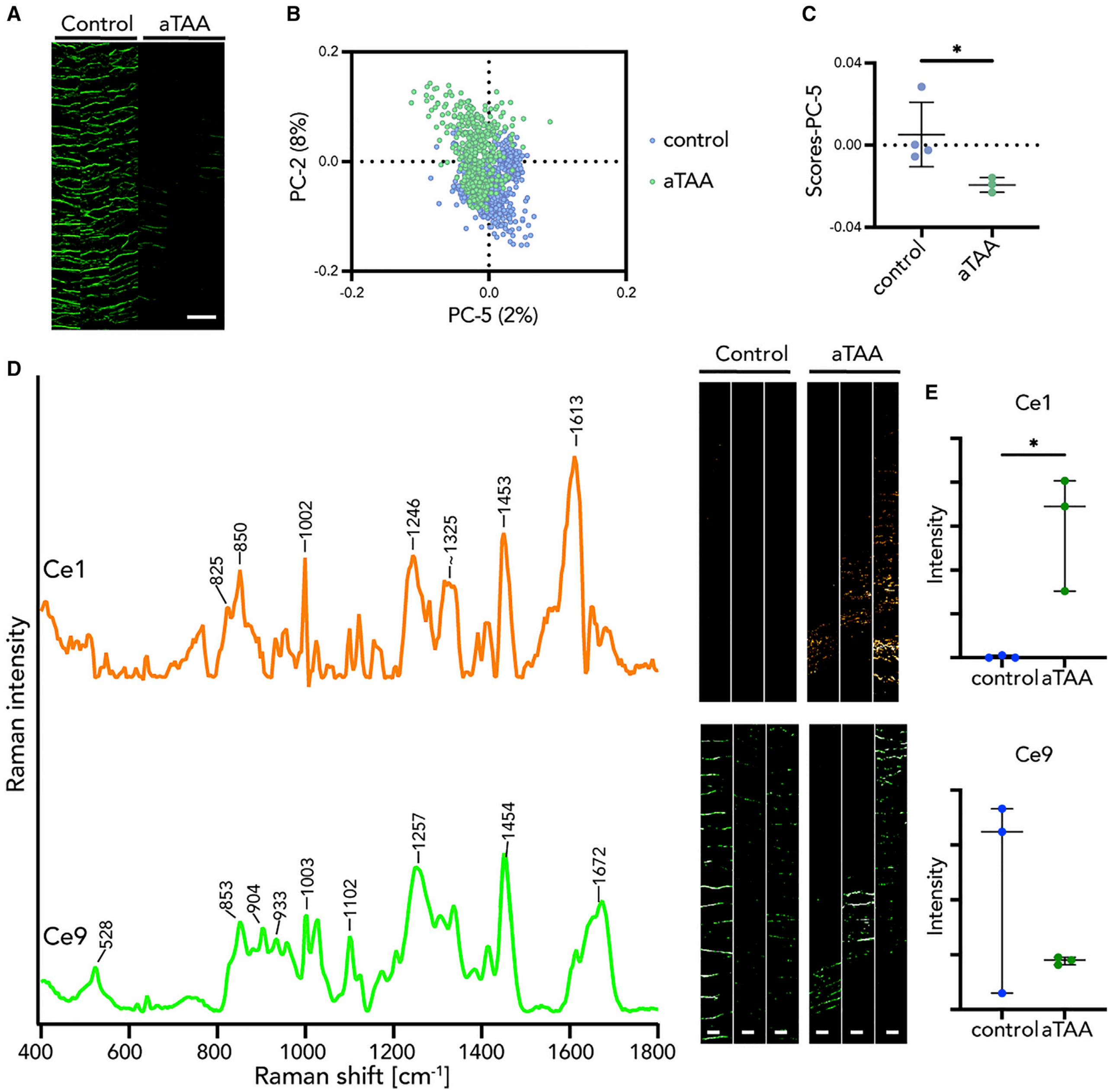


**Figure 5. Multivariate analysis (MVA) of elastic fibers revealed similar structural patterns in murine and human aneurysm tissues**
(A) Intensity distribution heatmaps of elastic fibers. Scale bar, 50 μm.
(B and C) PC-5 score values showed a separation between aTAA and human control.
(D and E) By MCR, the murine aneurysm-specific spectral marker Ce1 (orange) was confirmed in human TAA tissue and could not be found in human control tissues, whereas the native elastic fiber signature Ce9 (green) was localized in both groups.
Scale bars, 20 μm. N = 3, data points represent mean score (C) or intensity (E) values ± SD, non-paired t test (C and Ce9) or non-parametric Mann-Whitney test (Ce1) were performed; $^{*}p < 0.05$.
See also Figure S4 and Tables S1 and S2.

components. PCA and MCR enabled the extraction and discrimination of structural alterations on a submolecular level beyond species differences.

Raman microspectroscopy previously detected changes in the Raman signatures of fibrillin-1 microfibrils and elastic fiber networks in the skin of healthy mice and a murine model of Marfan syndrome.[28] In the present study, we demonstrated that the two identified signatures in elastic and collagen fibers have high potential as diagnostic biomarkers for aortic aneurysms. By increasing the components of Raman spectra and

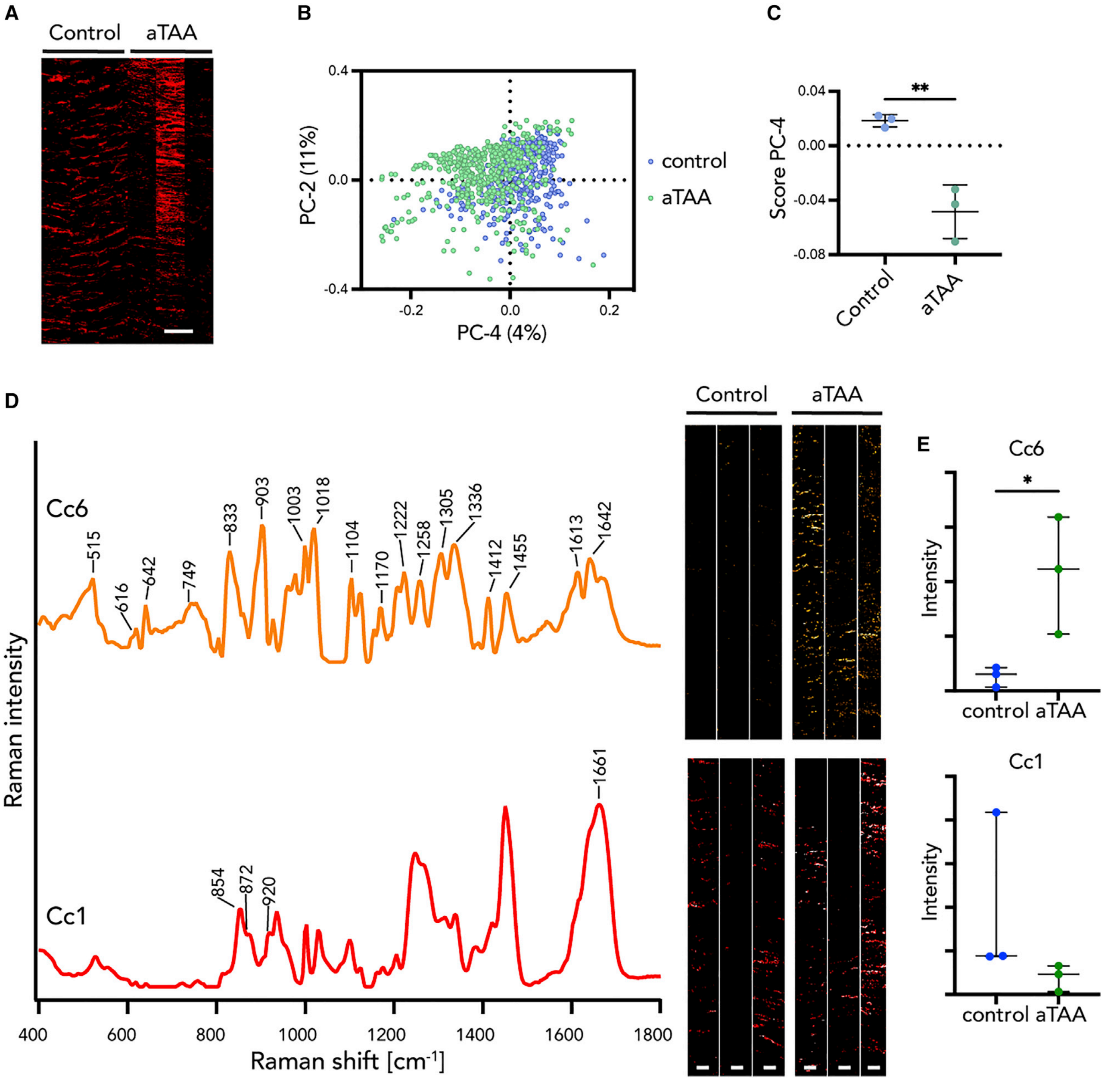


**Figure 6. MVA on collagen fibers revealed similar structural patterns in murine and human aneurysm tissues**

(A) Intensity distribution heatmaps of collagen fibers in human control and ascending thoracic aortic aneurysm (aTAA). Scale bar equals 50 μm.

(B and C) PCA showing a clustering between control and aneurysm in PC-4.

(D and E) MCR decomposed Raman spectra and imaging of murine Cc6 (aneurysm feature, orange) and Cc1 (native collagen feature, red) components applied to human control and TAA tissues indicate a significant difference in the aneurysm-specific signature (Cc6) but not in the native collagen signature (Cc1). Scale bars equal 5 μm. N = 3, data points are represented as mean score (C) or intensity (E) values ± SD, non-paired t tests were performed; $^{*}p < 0.05$, $^{**}p < 0.01$. See also Figure S4 and Tables S1 and S2.

improving the resolution, Raman imaging can quickly evaluate vessel wall integrity and biochemical components of the diseased vessel wall. Future research may explore other TAA models that develop in adulthood, including fibrillin-1 mutant mice (*Fbn1*$^{mgR/mgR}$) and apolipoprotein E-deficient mice.[55,56]

Recently, several applications for marker-independent Raman imaging have been reported; however, the imaging itself does not provide sufficient information to identify Raman marker spectra associated with cardiovascular diseases.[32,57,58] The *Fbln4*$^{SMKO}$ model recapitulates ascending aneurysms of cutis laxa type I, which is a rare disease. However, the spectral data

CellPress
OPEN ACCESS

obtained from *Fbln4*$^{SMKO}$ aortas remarkably depicted ECM changes in human aTAA, indicating the versatile application of *in vivo* experimental data to human conditions.

For clinical implementation, Raman spectroscopy probe techniques have already been reported in determining structural features in cancer, skin, and oral tissues.[59–61] Raman measurements are now used for rapid intraoperative diagnosis by stimulated Raman histology for brain tumors.[62–64] Combined with automated spectral decomposition methods, Raman techniques have the potential to extract target signals from big datasets without the need for extensive training of the end user. Fiberoptic confocal Raman probes have already been applied in preclinical setups. Wang et al.[65] used Raman endoscopy to measure epithelial tissues. Recently, fiberoptic Raman endoscopy has been used in patients with recurrent nasopharyngeal carcinoma to follow up on tumor progression.[66] The field of endoscopy is rapidly evolving, as the first robotic magnetic flexible endoscope systems are now available.[67] Therefore, fiberoptic Raman endoscopy for the aorta has the potential as a research tool as well as in a clinical setting. Furthermore, a combination of Raman measurements and artificial intelligence to identify molecular tissue patterns specific to (pre-)aneurysm will be a robust tool for modeling and monitoring the risk for aneurysm rupture/dissection.

### Limitations of study

We are aware that the number of human aTAA patient samples is relatively small and needs to be increased to translate the established method into clinical applications. At this stage, human samples served as a proof-of-principle approach to evaluate the relevance of the findings in the aneurysm mouse model. In addition, the character of the study is mainly readout oriented. We used this highly molecular-sensitive technique but focused on identifying an aneurysm-specific signature as a spectral marker rather than a complex biomolecular characterization of aneurysm tissue. Structural differences were mainly linked to Col I and elastic fibers; however, the availability of data on spectral references of elastic fiber substructures (e.g., tropoelastin, fibrillin, fibulins) remains restricted and needs to be further elaborated for mechanistic investigations on aneurysm formation.

## STAR★METHODS

Detailed methods are provided in the online version of this paper and include the following:

- KEY RESOURCES TABLE
- RESOURCE AVAILABILITY
  - ○ Lead contact
  - ○ Materials availability
  - ○ Data and code availability
- EXPERIMENTAL MODEL AND SUBJECT DETAILS
  - ○ Mice and tissue sections
  - ○ Human tissue sections
- METHOD DETAILS
  - ○ Immunofluorescence staining
  - ○ Histochemistry
  - ○ Raman measurements
  - ○ Raman imaging analysis
  - ○ Principal-component analysis (PCA)
  - ○ Multivariate curve resolution (MCR)
  - ○ Statistical analysis

## SUPPLEMENTAL INFORMATION

Supplemental information can be found online at https://doi.org/10.1016/j.xcrm.2021.100261.

## ACKNOWLEDGMENTS

This work was supported in part by MEXT KAKENHI (grant nos. JP 17H04289 and JP 20H03762) and SENSHIN Medical Research Foundation, Japan, to H.Y., as well as the Deutsche Forschungsgemeinschaft (INST 2388/64-1 and Germany's Excellence Strategy, EXC 2180-390900677), the Ministry of Science, Research, and the Arts of Baden-Württemberg (33-729.55-3/214 and SI-BW 01222-91, to K.S.-L.), and the State Ministry of Baden-Württemberg for Economic Affairs, Labour and Housing Construction. M.A. was supported by JST, PRESTO (grant no. JPMJPR1671), Japan.

## AUTHOR CONTRIBUTIONS

The experiments were designed by K.S., K.S.-L., and H.Y. The Raman experiments were measured by K.S., J.A. (murine samples), and J.M. (human samples). The data analysis was executed by K.S., J.M., and M.A. TCA was analyzed by K.S. and J.M. PCA was mainly analyzed by J.M. MCR was mainly analyzed by K.S. and M.A. The murine samples were prepared by K.S., Y.Y., and H.Y. The human samples were provided by B.R. K.S. and J.M. wrote the manuscript, which was revised by E.M.B., M.A., K.S.-L., and H.Y. All of the authors commented on the paper.

## DECLARATION OF INTERESTS

The authors declare no competing interests.

## INCLUSION AND DIVERSITY

One or more of the authors of this paper self-identifies as an underrepresented ethnic minority in science. It takes all kinds of minds to uncover new scientific discoveries.

Received: April 26, 2020
Revised: January 29, 2021
Accepted: April 6, 2021
Published: April 28, 2021

## REFERENCES

1. Isselbacher, E.M. (2014). Trends in thoracic aortic aneurysms and dissection: out of the shadows and into the light. Circulation *130*, 2267–2268.
2. Norman, P.E., and Powell, J.T. (2010). Site specificity of aneurysmal disease. Circulation *121*, 560–568.
3. Trimarchi, S., Jonker, F.H., Hutchison, S., Isselbacher, E.M., Pape, L.A., Patel, H.J., Froehlich, J.B., Muhs, B.E., Rampoldi, V., Grassi, V., et al. (2011). Descending aortic diameter of 5.5 cm or greater is not an accurate predictor of acute type B aortic dissection. J. Thorac. Cardiovasc. Surg. *142*, e101–e107.
4. Dietz, H.C., and Pyeritz, R.E. (1995). Mutations in the human gene for fibrillin-1 (FBN1) in the Marfan syndrome and related disorders. Hum. Mol. Genet. *4*, 1799–1809.
5. Pope, F.M., Martin, G.R., Lichtenstein, J.R., Penttinen, R., Gerson, B., Rowe, D.W., and McKusick, V.A. (1975). Patients with Ehlers-Danlos syndrome type IV lack type III collagen. Proc. Natl. Acad. Sci. USA *72*, 1314–1316.

6. Yeowell, H.N., Walker, L.C., Farmer, B., Heikkinen, J., and Myllyla, R. (2000). Mutational analysis of the lysyl hydroxylase 1 gene (PLOD) in six unrelated patients with Ehlers-Danlos syndrome type VI: prenatal exclusion of this disorder in one family. Hum. Mutat. *16*, 90.

7. Dasouki, M., Markova, D., Garola, R., Sasaki, T., Charbonneau, N.L., Sakai, L.Y., and Chu, M.L. (2007). Compound heterozygous mutations in fibulin-4 causing neonatal lethal pulmonary artery occlusion, aortic aneurysm, arachnodactyly, and mild cutis laxa. Am. J. Med. Genet. A. *143A*, 2635–2641.

8. Renard, M., Holm, T., Veith, R., Callewaert, B.L., Adès, L.C., Baspinar, O., Pickart, A., Dasouki, M., Hoyer, J., Rauch, A., et al. (2010). Altered TGFbeta signaling and cardiovascular manifestations in patients with autosomal recessive cutis laxa type I caused by fibulin-4 deficiency. Eur. J. Hum. Genet. *18*, 895–901.

9. Isselbacher, E.M., Lino Cardenas, C.L., and Lindsay, M.E. (2016). Hereditary Influence in Thoracic Aortic Aneurysm and Dissection. Circulation *133*, 2516–2528.

10. Milewicz, D.M., Trybus, K.M., Guo, D.C., Sweeney, H.L., Regalado, E., Kamm, K., and Stull, J.T. (2017). Altered Smooth Muscle Cell Force Generation as a Driver of Thoracic Aortic Aneurysms and Dissections. Arterioscler. Thromb. Vasc. Biol. *37*, 26–34.

11. Karimi, A., and Milewicz, D.M. (2016). Structure of the Elastin-Contractile Units in the Thoracic Aorta and How Genes That Cause Thoracic Aortic Aneurysms and Dissections Disrupt This Structure. Can. J. Cardiol. *32*, 26–34.

12. Cikach, F.S., Koch, C.D., Mead, T.J., Galatioto, J., Willard, B.B., Emerton, K.B., Eagleton, M.J., Blackstone, E.H., Ramirez, F., Roselli, E.E., and Apte, S.S. (2018). Massive aggrecan and versican accumulation in thoracic aortic aneurysm and dissection. JCI Insight *3*, 97167.

13. Petsophonsakul, P., Furmanik, M., Forsythe, R., Dweck, M., Schurink, G.W., Natour, E., Reutelingsperger, C., Jacobs, M., Mees, B., and Schurgers, L. (2019). Role of Vascular Smooth Muscle Cell Phenotypic Switching and Calcification in Aortic Aneurysm Formation. Arterioscler. Thromb. Vasc. Biol. *39*, 1351–1368.

14. Huang, J., Davis, E.C., Chapman, S.L., Budatha, M., Marmorstein, L.Y., Word, R.A., and Yanagisawa, H. (2010). Fibulin-4 deficiency results in ascending aortic aneurysms: a potential link between abnormal smooth muscle cell phenotype and aneurysm progression. Circ. Res. *106*, 583–592.

15. Spencer, J.A., Hacker, S.L., Davis, E.C., Mecham, R.P., Knutsen, R.H., Li, D.Y., Gerard, R.D., Richardson, J.A., Olson, E.N., and Yanagisawa, H. (2005). Altered vascular remodeling in fibulin-5-deficient mice reveals a role of fibulin-5 in smooth muscle cell proliferation and migration. Proc. Natl. Acad. Sci. USA *102*, 2946–2951.

16. Adiguzel, E., Hou, G., Sabatini, P.J., and Bendeck, M.P. (2013). Type VIII collagen signals via β1 integrin and RhoA to regulate MMP-2 expression and smooth muscle cell migration. Matrix Biol. *32*, 332–341.

17. Papke, C.L., and Yanagisawa, H. (2014). Fibulin-4 and fibulin-5 in elastogenesis and beyond: insights from mouse and human studies. Matrix Biol. *37*, 142–149.

18. Iascone, M., Sana, M.E., Pezzoli, L., Bianchi, P., Marchetti, D., Fasolini, G., Sadou, Y., Locatelli, A., Fabiani, F., Mangili, G., and Ferrazzi, P. (2012). Extensive arterial tortuosity and severe aortic dilation in a newborn with an EFEMP2 mutation. Circulation *126*, 2764–2768.

19. Yanagisawa, H., Davis, E.C., Starcher, B.C., Ouchi, T., Yanagisawa, M., Richardson, J.A., and Olson, E.N. (2002). Fibulin-5 is an elastin-binding protein essential for elastic fibre development in vivo. Nature *415*, 168–171.

20. Papke, C.L., Tsunezumi, J., Ringuette, L.J., Nagaoka, H., Terajima, M., Yamashiro, Y., Urquhart, G., Yamauchi, M., Davis, E.C., and Yanagisawa, H. (2015). Loss of fibulin-4 disrupts collagen synthesis and maturation: implications for pathology resulting from EFEMP2 mutations. Hum. Mol. Genet. *24*, 5867–5879.

21. Horiguchi, M., Inoue, T., Ohbayashi, T., Hirai, M., Noda, K., Marmorstein, L.Y., Yabe, D., Takagi, K., Akama, T.O., Kita, T., et al. (2009). Fibulin-4 conducts proper elastogenesis via interaction with cross-linking enzyme lysyl oxidase. Proc. Natl. Acad. Sci. USA *106*, 19029–19034.

22. Choudhury, N., Bouchot, O., Rouleau, L., Tremblay, D., Cartier, R., Butany, J., Mongrain, R., and Leask, R.L. (2009). Local mechanical and structural properties of healthy and diseased human ascending aorta tissue. Cardiovasc. Pathol. *18*, 83–91.

23. Camp, C.H., Jr., Lee, Y.J., Heddleston, J.M., Hartshorn, C.M., Hight Walker, A.R., Rich, J.N., Lathia, J.D., and Cicerone, M.T. (2014). High-Speed Coherent Raman Fingerprint Imaging of Biological Tissues. Nat. Photonics *8*, 627–634.

24. Smith, R., Wright, K.L., and Ashton, L. (2016). Raman spectroscopy: an evolving technique for live cell studies. Analyst (Lond.) *141*, 3590–3600.

25. Frushour, B.G., and Koenig, J.L. (1975). Raman scattering of collagen, gelatin, and elastin. Biopolymers *14*, 379–391.

26. Pudlas, M., Koch, S., Bolwien, C., Thude, S., Jenne, N., Hirth, T., Walles, H., and Schenke-Layland, K. (2011). Raman spectroscopy: a noninvasive analysis tool for the discrimination of human skin cells. Tissue Eng. Part C Methods *17*, 1027–1040.

27. Qian, X., Peng, X.H., Ansari, D.O., Yin-Goen, Q., Chen, G.Z., Shin, D.M., Yang, L., Young, A.N., Wang, M.D., and Nie, S. (2008). In vivo tumor targeting and spectroscopic detection with surface-enhanced Raman nanoparticle tags. Nat. Biotechnol. *26*, 83–90.

28. Brauchle, E., Bauer, H., Fernes, P., Zuk, A., Schenke-Layland, K., and Sengle, G. (2017). Raman microspectroscopy as a diagnostic tool for the non-invasive analysis of fibrillin-1 deficiency in the skin and in the in vitro skin models. Acta Biomater. *52*, 41–48.

29. Huang, Z., McWilliams, A., Lui, H., McLean, D.I., Lam, S., and Zeng, H. (2003). Near-infrared Raman spectroscopy for optical diagnosis of lung cancer. Int. J. Cancer *107*, 1047–1052.

30. Spiers, R.M., Marzi, J., Brauchle, E.M., Cross, S.E., Vaughan, R.H., Bateman, P.A., Hughes, S.J., Schenke-Layland, K., and Johnson, P.R.V. (2019). Donor age significantly influences the Raman spectroscopic biomolecular fingerprint of human pancreatic extracellular matrix proteins following collagenase-based digestion. Acta Biomater. *99*, 269–283.

31. Chaichi, A., Prasad, A., and Gartia, M.R. (2018). Raman Spectroscopy and Microscopy Applications in Cardiovascular Diseases: From Molecules to Organs. Biosensors (Basel) *8*, E107.

32. You, A.Y.F., Bergholt, M.S., St-Pierre, J.P., Kit-Anan, W., Pence, I.J., Chester, A.H., Yacoub, M.H., Bertazzo, S., and Stevens, M.M. (2017). Raman spectroscopy imaging reveals interplay between atherosclerosis and medial calcification in the human aorta. Sci. Adv. *3*, e1701156.

33. Marzi, J., Brauchle, E.M., Schenke-Layland, K., and Rolle, M.W. (2019). Non-invasive functional molecular phenotyping of human smooth muscle cells utilized in cardiovascular tissue engineering. Acta Biomater. *89*, 193–205.

34. Movasaghi, Z., Rehman, S., and Rehman, I.U. (2007). Raman spectroscopy of biological tissues. Appl. Spectrosc. Rev. *42*, 493–541.

35. Frank, C.J., McCreery, R.L., and Redd, D.C.B. (1995). Raman spectroscopy of normal and diseased human breast tissues. Anal. Chem. *67*, 777–783.

36. Cheng, W.T., Liu, M.T., Liu, H.N., and Lin, S.Y. (2005). Micro-Raman spectroscopy used to identify and grade human skin pilomatrixoma. Microsc. Res. Tech. *68*, 75–79.

37. Ellis, R., Green, E., and Winlove, C.P. (2009). Structural analysis of glycosaminoglycans and proteoglycans by means of Raman microspectrometry. Connect. Tissue Res. *50*, 29–36.

38. Notingher, I., Green, C., Dyer, C., Perkins, E., Hopkins, N., Lindsay, C., and Hench, L.L. (2004). Discrimination between ricin and sulphur mustard toxicity in vitro using Raman spectroscopy. J. R. Soc. Interface *1*, 79–90.

39. Sigurdsson, S., Philipsen, P.A., Hansen, L.K., Larsen, J., Gniadecka, M., and Wulf, H.C. (2004). Detection of skin cancer by classification of Raman spectra. IEEE Trans. Biomed. Eng. *51*, 1784–1793.

40. Shetty, G., Kendall, C., Shepherd, N., Stone, N., and Barr, H. (2006). Raman spectroscopy: elucidation of biochemical changes in carcinogenesis of oesophagus. Br. J. Cancer *94*, 1460–1464.

41. Sajan, D., Binoy, J., Pradeep, B., Venkata Krishna, K., Kartha, V.B., Hubert Joe, I., and Jayakumar, V.S. (2004). NIR-FT Raman and infrared spectra and ab initio computations of glycinium oxalate. Spectrochim. Acta A Mol. Biomol. Spectrosc. *60*, 173–180.

42. Stone, N., Kendall, C., Shepherd, N., Crow, P., and Barr, H. (2002). Near-infrared Raman spectroscopy for the classification of epithelial pre-cancers and cancers. J. Raman Spectrosc. *33*, 564–573.

43. Stone, N., Kendall, C., Smith, J., Crow, P., and Barr, H. (2004). Raman spectroscopy for identification of epithelial cancers. Faraday Discuss. *126*, 141–157, discussion 169–183.

44. Hanlon, E.B., Manoharan, R., Koo, T.W., Shafer, K.E., Motz, J.T., Fitzmaurice, M., Kramer, J.R., Itzkan, I., Dasari, R.R., and Feld, M.S. (2000). Prospects for in vivo Raman spectroscopy. Phys. Med. Biol. *45*, R1–R59.

45. Li, D.Y., Brooke, B., Davis, E.C., Mecham, R.P., Sorensen, L.K., Boak, B.B., Eichwald, E., and Keating, M.T. (1998). Elastin is an essential determinant of arterial morphogenesis. Nature *393*, 276–280.

46. Tuma, R. (2005). Raman spectroscopy of proteins: from peptides to large assemblies. J. Raman Spectrosc. *36*, 307–319.

47. Black, K.M., Masuzawa, A., Hagberg, R.C., Khabbaz, K.R., Trovato, M.E., Rettagliati, V.M., Bhasin, M.K., Dillon, S.T., Libermann, T.A., Toumpoulis, I.K., et al. (2013). Preliminary biomarkers for identification of human ascending thoracic aortic aneurysm. J. Am. Heart Assoc. *2*, e000138.

48. Wang, Q., Ding, Y., Song, P., Zhu, H., Okon, I., Ding, Y.N., Chen, H.Z., Liu, D.P., and Zou, M.H. (2017). Tryptophan-Derived 3-Hydroxyanthranilic Acid Contributes to Angiotensin II-Induced Abdominal Aortic Aneurysm Formation in Mice In Vivo. Circulation *136*, 2271–2283.

49. Bergholt, M.S., St-Pierre, J.P., Offeddu, G.S., Parmar, P.A., Albro, M.B., Puetzer, J.L., Oyen, M.L., and Stevens, M.M. (2016). Raman Spectroscopy Reveals New Insights into the Zonal Organization of Native and Tissue-Engineered Articular Cartilage. ACS Cent. Sci. *2*, 885–895.

50. Mithieux, S.M., Rasko, J.E., and Weiss, A.S. (2004). Synthetic elastin hydrogels derived from massive elastic assemblies of self-organized human protein monomers. Biomaterials *25*, 4921–4927.

51. Debelle, L., Alix, A.J., Wei, S.M., Jacob, M.P., Huvenne, J.P., Berjot, M., and Legrand, P. (1998). The secondary structure and architecture of human elastin. Eur. J. Biochem. *258*, 533–539.

52. Azeloglu, E.U., Albro, M.B., Thimmappa, V.A., Ateshian, G.A., and Costa, K.D. (2008). Heterogeneous transmural proteoglycan distribution provides a mechanism for regulating residual stresses in the aorta. Am. J. Physiol. Heart Circ. Physiol. *294*, H1197–H1205.

53. Suna, G., Wojakowski, W., Lynch, M., Barallobre-Barreiro, J., Yin, X., Mayr, U., Baig, F., Lu, R., Fava, M., Hayward, R., et al. (2018). Extracellular Matrix Proteomics Reveals Interplay of Aggrecan and Aggrecanases in Vascular Remodeling of Stented Coronary Arteries. Circulation *137*, 166–183.

54. Roccabianca, S., Bellini, C., and Humphrey, J.D. (2014). Computational modelling suggests good, bad and ugly roles of glycosaminoglycans in arterial wall mechanics and mechanobiology. J. R. Soc. Interface *11*, 20140397.

55. Daugherty, A., and Cassis, L.A. (2004). Mouse models of abdominal aortic aneurysms. Arterioscler. Thromb. Vasc. Biol. *24*, 429–434.

56. Bellini, C., Bersi, M.R., Caulk, A.W., Ferruzzi, J., Milewicz, D.M., Ramirez, F., Rifkin, D.B., Tellides, G., Yanagisawa, H., and Humphrey, J.D. (2017). Comparison of 10 murine models reveals a distinct biomechanical phenotype in thoracic aortic aneurysms. J. R. Soc. Interface *14*, 20161036.

57. Wei, L., Chen, Z., Shi, L., Long, R., Anzalone, A.V., Zhang, L., Hu, F., Yuste, R., Cornish, V.W., and Min, W. (2017). Super-multiplex vibrational imaging. Nature *544*, 465–470.

58. Vogt, N. (2019). Super-resolution Raman imaging. Nat. Methods *16*, 1202.

59. Stevens, O., Iping Petterson, I.E., Day, J.C.C., and Stone, N. (2016). Developing fibre optic Raman probes for applications in clinical spectroscopy. Chem. Soc. Rev. *45*, 1919–1934.

60. Jermyn, M., Mok, K., Mercier, J., Desroches, J., Pichette, J., Saint-Arnaud, K., Bernstein, L., Guiot, M.C., Petrecca, K., and Leblond, F. (2015). Intraoperative brain cancer detection with Raman spectroscopy in humans. Sci. Transl. Med. *7*, 274ra19.

61. Carvalho, L.F.C.S., Nogueira, M.S., Bhattacharjee, T., Neto, L.P.M., Daun, L., Mendes, T.O., Rajasekaran, R., Chagas, M., Martin, A.A., and Soares, L.E.S. (2019). In vivo Raman spectroscopic characteristics of different sites of the oral mucosa in healthy volunteers. Clin. Oral Investig. *23*, 3021–3031.

62. Orringer, D.A., Pandian, B., Niknafs, Y.S., Hollon, T.C., Boyle, J., Lewis, S., Garrard, M., Hervey-Jumper, S.L., Garton, H.J.L., Maher, C.O., et al. (2017). Rapid intraoperative histology of unprocessed surgical specimens via fibre-laser-based stimulated Raman scattering microscopy. Nat. Biomed. Eng. *1*, 0027.

63. Hollon, T.C., Lewis, S., Pandian, B., Niknafs, Y.S., Garrard, M.R., Garton, H., Maher, C.O., McFadden, K., Snuderl, M., Lieberman, A.P., et al. (2018). Rapid Intraoperative Diagnosis of Pediatric Brain Tumors Using Stimulated Raman Histology. Cancer Res. *78*, 278–289.

64. Livermore, L.J., Isabelle, M., Bell, I.M., Scott, C., Walsby-Tickle, J., Gannon, J., Plaha, P., Vallance, C., and Ansorge, O. (2019). Rapid intraoperative molecular genetic classification of gliomas using Raman spectroscopy. Neurooncol. Adv. *1*, vdz008.

65. Wang, J., Bergholt, M.S., Zheng, W., and Huang, Z. (2013). Development of a beveled fiber-optic confocal Raman probe for enhancing in vivo epithelial tissue Raman measurements at endoscopy. Opt. Lett. *38*, 2321–2323.

66. Shu, C., Zheng, W., Lin, K., Lim, C., and Huang, Z. (2021). Label-Free Follow-Up Surveying of Post-Treatment Efficacy and Recurrence in Nasopharyngeal Carcinoma Patients with Fiberoptic Raman Endoscopy. Anal. Chem. *93*, 2053–2061.

67. Martin, J.W., Scaglioni, B., Norton, J.C., Subramanian, V., Arezzo, A., Obstein, K.L., and Valdastri, P. (2020). Enabling the future of colonoscopy with intelligent and autonomous magnetic manipulation. Nat. Mach. Intell. *2*, 595–606.

68. Ando, M., and Hamaguchi, H.O. (2014). Molecular component distribution imaging of living cells by multivariate curve resolution analysis of space-resolved Raman spectra. J. Biomed. Opt. *19*, 0110161.

69. Arai, S., Shelton, J.M., Chen, M., Bradley, M.N., Castrillo, A., Bookout, A.L., Mak, P.A., Edwards, P.A., Mangelsdorf, D.J., Tontonoz, P., and Miyazaki, T. (2005). A role for the apoptosis inhibitory factor AIM/Spalpha/Api6 in atherosclerosis development. Cell Metab. *1*, 201–213.

70. Marzi, J., Biermann, A.C., Brauchle, E.M., Brockbank, K.G.M., Stock, U.A., and Schenke-Layland, K. (2019). Marker-Independent In Situ Quantitative Assessment of Residual Cryoprotectants in Cardiac Tissues. Anal. Chem. *91*, 2266–2272.

71. Zbinden, A., Marzi, J., Schlünder, K., Probst, C., Urbanczyk, M., Black, S., Brauchle, E.M., Layland, S.L., Kraushaar, U., Duffy, G., et al. (2020). Non-invasive marker-independent high content analysis of a microphysiological human pancreas-on-a-chip model. Matrix Biol. *85-86*, 205–220.

72. Hoyer, P.O. (2004). Non-negative matrix factorization with sparseness constraints. J. Mach. Learn. Res. *5*, 1457–1469.

# STAR★METHODS

## KEY RESOURCES TABLE

| REAGENT OR RESOURCE | SOURCE | IDENTIFIER |
|---|---|---|
| Antibodies | | |
| Rabbit anti-mouse collagen type I polyclonal antibody | Millipore | Cat#: AB765P, RRID:AB_92259 |
| Rabbit anti-mouse versican (GAG beta domain) polyclonal antibody | Millipore | Cat#: AB1033, RRID: AB_90462 |
| Anti-aggrecan polyclonal antibody | Millipore | Cat#: AB1031, RRID:AB_90460 |
| Alexa Fluor 546, anti-mouse | Thermo Fisher Scientific | Cat# A-11003, RRID:AB_2534071) |
| Alexa Fluor 546, anti-rabbit | Thermo Fisher Scientific | Cat# A-11035, RRID:AB_2534093 |
| VECTASHIELD Mounting Medium antibody | Vector Laboratories | Cat#: H-1000, RRID:AB_2336789 |
| Biological samples | | |
| Human aorta block | New York University Langone Medical Center | N/A |
| Chemicals, peptides, and recombinant proteins | | |
| Aggrecan from bovine articular cartilage | Millipore Sigma | Cat#: A1960 |
| Mouse VCAN / versican protein (Recombinat His, N-Terminal) (aa3058-3299) | LifeSpan BioSciences Inc. | Cat#: LS-G14317-10 |
| Oil Red O | FUJIFILM Wako Pure Chemical Corporation | Cat#: 1320-06-5 |
| Alcian Blue Solution | FUJIFILM Wako Pure Chemical Corporation | Cat#: 75881-23-1 |
| Experimental models: organisms/strains | | |
| Mouse: C57BL/6J | The Jackson Laboratory | JAX: 000664 |
| Mouse: 129S6/SvEVTac | Taconic Biosciences | 129SVE |
| Mouse: Fbln4$^{SMKO}$ | Huang et al., 2010[14] | N/A |
| Mouse: Fbln5$^{KO}$ | Yanagisawa et al., 2002[19] | N/A |
| Mouse: Eln$^{KO}$ | Li et al., 1998[45] | N/A |
| Software and algorithms | | |
| Igor Pro | Wave Metrics | https://www.wavemetrics.com/products/igorpro/igorpro.htm RRID:SCR_000325 |
| Python | Python Software | https://www.python.org/, RRID:SCR_008394 |
| MCR-ALS | Ando and Hamaguchi, 2014[68] | https://github.com/mshrAndo/PyMCR |
| Project FIVE 5.0 software | WiTec GmbH | https://www.witec.de/jp/products/accessories/software-witec-suite/ |
| GraphPad Prism 8 | GraphPad Software | RRID:SCR_002798 https://www.graphpad.com/ |
| Unscrambler X | CAMO Software | http://www.stjapan.co.jp/products/1658 |

## RESOURCE AVAILABILITY

### Lead contact

Further information and requests for resources should be directed to and will be fulfilled by the lead contact, Hiromi Yanagisawa (hkyanagisawa@tara.tsukuba.ac.jp).

### Materials availability

This study did not generate unique reagents. Genetic mouse models will be available upon request after completion of a Materials Transfer Agreement.

## Data and code availability

The data of this study are available without restriction. MCR bioinformatics code can be found online (https://github.com/mshrAndo/PyMCR).

## EXPERIMENTAL MODEL AND SUBJECT DETAILS

### Mice and tissue sections

*Fbln5*$^{KO}$, *Fbln4*$^{SMKO}$, and *Eln* null (*Eln*$^{KO}$) mice were previously described and maintained on a C57/Bl6;129SvEv background.[14,19,45] Animals at 1 to 3 months of age were used as adult mice for *Fbln5*$^{KO}$, *Fbln4*$^{SMKO}$, and wild-type (WT). For *Eln*$^{KO}$ and WT control, post-natal day (P) 1 neonates were used. Six to seven mice per genotype of adult mice for *Fbln5*$^{KO}$, *Fbln4*$^{SMKO}$, WT and thee mice per genotype of P1 mice for *Eln*$^{KO}$ and WT were used in all experiments and aortic tissues were harvested, embedded in Tissue Tek ® O.C.T. medium (Tissue Tek, Sakura), and snap-frozen in liquid nitrogen or embedded in paraffin. Mice were housed in the specific pathogen-free condition under a 12 h/12 h light/dark cycle. All animal protocols were approved by the Institutional Animal Experiment Committee of the University of Tsukuba.

### Human tissue sections

Human ascending aorta aTAA samples were collected from open surgical repairs. Control aortic tissues were obtained from multi-organ donors confirmed as brain-dead. aTAA and control aortic tissues were analyzed for three different patients and donors. All studies were approved by New York University Langone Medical Center Institutional Review Board (IRB). Tissues were formalin-fixed and paraffin embedded prior to sectioning and Raman microscopy analysis.

## METHOD DETAILS

### Immunofluorescence staining

Ten-micrometer cross sections of the murine aorta prepared in Tissue Tek® O.C.T frozen blocks were fixed with 4% paraformaldehyde (PFA) for 10 min at room temperature (RT). Sections were incubated with blocking buffer containing 5% normal goat serum in PBS and permeabilized with 0.1% Triton-X for 30 min at RT. Primary antibodies used were: anti-mouse collagen type I antibody (1:100; AB765P, Merck Millipore), rabbit anti-mouse versican (GAG beta domain) polyclonal antibody (1:200; AB1033, Chemicon international), rabbit anti-aggrecan polyclonal antibody (1:200; AB1031, Chemicon international). All antibodies were diluted in 5% BSA with 0.1% Triton-X in PBS and incubated overnight at 4°C. Secondary antibodies (Alexa 546 mouse or rabbit) were incubated for 1 hr at RT. Vectashield with DAPI (Vector Laboratories) was used to mount the slides and samples were analyzed with a laser scanning microscope LSM 710 (Zeiss GmbH). At least three mice per genotype were used for analysis.

### Histochemistry

Paraffin-embedded sections were stained with Alcian blue for glycosaminoglycans following the standard protocol.[20] Cross sections of aortas from Tissue Tek® O.C.T frozen block were fixed with 4% PFA for 10 min at RT and stained with Oil Red O (ORO) (Wako, Japan) to visualize lipids.[69] Three mice per genotype were used for analysis.

### Raman measurements

Raman measurements were performed with a confocal Raman microscope (WiTec alpha 300 R, WiTec GmbH, Ulm, Germany) equipped with a green laser (532 nm) as described previously.[70] Cross sections of aortic tissues were rinsed with PBS and a 63x Apochromat water dipping objective (N.A. 1.0; Carl Zeiss GmbH) was utilized for data acquisition. Large area scans were performed on areas of 100 × 200 μm with 2 × 2 μm pixel resolution and acquisition time of 0.25 s per spectrum. High-resolution scans were performed on areas of 10 × 150 μm with 1 × 1 μm pixel resolution and 0.5 s acquisition time per spectrum. Laser power was set to 50 mW. Reference spectra were obtained from lyophilized aggrecan from bovine articular cartilage (A1960, Sigma-Aldrich/Merck, Darmstadt Germany) and recombinant lyophilized murine versican (LG-G14317, LifeSpan BioSciences Inc., Seattle, USA). Single spectra were acquired with a laser power of 50 mW and an acquisition time of 5 s. For the murine tissues, samples were prepared with as frozen sections, washed, and kept under PBS during the measurements. Seven mice per genotype were used and at least three areas were examined per tissue sample. For the human tissues, samples were deparaffinized, rehydrated, and kept under PBS during the entire measurement. High-resolution scans of an area of 50 × 500 μm were acquired at a pixel resolution of 1 × 1 μm, an integration time of 0.5 s, and a laser power of 50 mW. Three control and three aTAA samples were obtained, and at least three areas per section were analyzed.

### Raman imaging analysis

Spectral data were pre-processed by cosmic ray removal and background subtraction. The spectral maps were analyzed by True Component Analysis (TCA), a tool from Project FIVE 5.0 software (WiTec GmbH) that identified different spectral information within the dataset, as previously described.[71] Briefly, TCA is a non-negative matrix factorization-based algorithm that defines similar spectra as the same component and allows for the generation of color-coded intensity distribution heatmaps of the identified

structures. TCA components identified in the murine WT aortas were applied as reference signatures for the TCA analysis of the KO animals as well as the human tissues.

### Principal-component analysis (PCA)

Raman spectral datasets were extracted for elastic and collagen fiber components identified by TCA. The data were normalized and the Raman shift range was cropped to the fingerprint region between 400 and 1800 $cm^{-1}$. The Unscrambler X10.5 (CAMO Software, AS, Oslo, Norway) was applied to perform principal component analysis (PCA) using NIPALS algorithm. PCA is commonly implemented in chemometrics and allows for the identification and interpretation of spectral differences within a Raman dataset.[28,33] Briefly, in PCA, spectral information is described by vectors, so-called principal components (PCs). PCA results are visualized by a scores plot, where one PC is plotted against another, with every spectrum represented as an individual dot. The corresponding PC loadings plot displays peaks that have a great impact on the scores values and allows for the interpretation of molecular differences among the groups.[28,43] The average of the score values in each animal was calculated and used for statistical analysis.

### Multivariate curve resolution (MCR)

Multivariate curve resolution by alternating least-squares (MCR-ALS) was effectively used to extract chemically interpretable spectra.[68] In MCR-ALS, an original data matrix (***A***) can be decomposed into two matrices—***W***, spectral components, and ***H***, their intensity contribution profiles—as given below.

$$\boldsymbol{A}_{m \times n} = \boldsymbol{W}_{m \times k}\boldsymbol{H}_{k \times n} + \boldsymbol{E}_{m \times n}$$

Here ***E*** is the error matrix. In the present study, the elastic fiber and collagen fiber datasets were preselected by TCA and whole area intensity normalized elastic fiber/collagen fiber datasets were used as ***A***. In the optimization calculation, non-negativity constraints ($W_{ij} \geq 0$ and $H_{ij} \geq 0$) are introduced in order to conduct a physically adaptable decomposition; Raman scattering intensities and molecular concentrations are always non-negative. Furthermore, in the present study, $l_1$-norm regularization (Lasso regression) was applied to enable the extraction of specific spectral components, via sparser matrix factorization, that contribute to the difference among samples, which were mice aortas in WT, *Fbln5*$^{KO}$, and *Fbln4*$^{SMKO}$, and human aortas in control and aTAA patients.[72] Under these constraints, using singular value decomposition (SVD)-based initialization (applied to fingerprint region between 400 to 1800 $cm^{-1}$), MCR-ALS optimization was performed by minimizing $(\|A - WH\|_2 + \|\lambda H\|_1)$. The hyperparameter $\lambda$ for Lasso regression was determined as 0.002 by cross validation. For murine analysis, SVD initialization was utilized to identify MCR components, and for human analysis, the results of murine MCR analysis were utilized as initial spectra. All optimization calculations were performed using an in-house program written in Python code.[68] The average intensity of $\boldsymbol{h}_i$ vector for each mapping image was calculated and used for the statistical analysis.

### Statistical analysis

Data were represented as mean ± SD. Statistical analysis was performed using Prism 9 software (GraphPad, La Jolla, CA, USA). Shapiro-Wilk tests were conducted to examine whether the data followed normal distribution. If the data followed normal distribution, statistical significance was determined by unpaired or paired t test for two-group comparisons and one-way/two-way analysis of variance (ANOVA) for comparison among three or more groups followed by Bonferroni's correction for multiple comparison tests. If the normality assumption was violated, nonparametric tests (Mann-Whitney or Kruskal-Wallis) were conducted. Data were considered statistically significant for p values of 0.05 or less.

Cell Reports Medicine, Volume *2*

## Supplemental information

# Raman microspectroscopy and Raman imaging reveal biomarkers specific for thoracic aortic aneurysms

**Kaori Sugiyama, Julia Marzi, Julia Alber, Eva M. Brauchle, Masahiro Ando, Yoshito Yamashiro, Bhama Ramkhelawon, Katja Schenke-Layland, and Hiromi Yanagisawa**

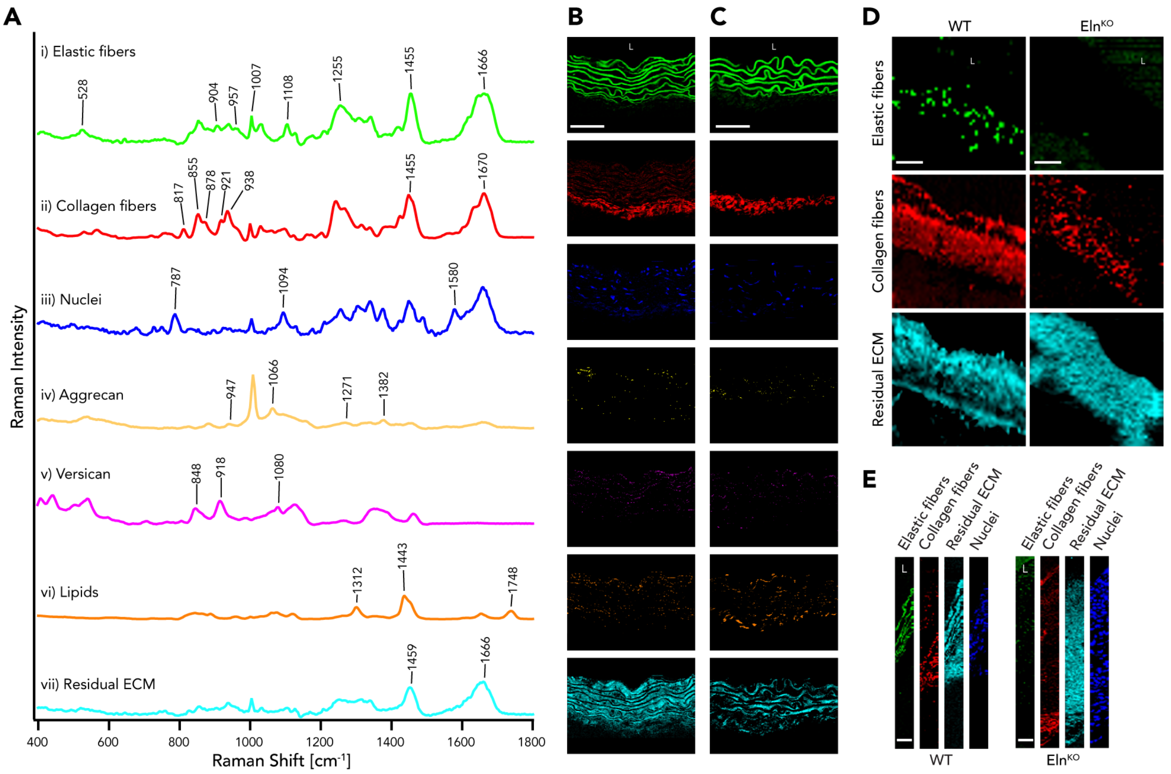


**Supplemental Figure S1**. **Raman imaging of cross sections of murine ascending and descending aortas by true component analysis (TCA), and confirmation of the specificity of the elastic fiber reference spectrum. Related to Figure 1.** (A) Identified spectral components correlate to elastic fibers (green), collagen fibers (red), nuclei (blue), aggrecan (yellow), versican (pink), lipids (orange), and residual ECM (cyan). (B) Corresponding intensity distribution heatmaps for each component of ascending aorta. Scale bars equal 50 µm. (C) Corresponding intensity distribution heatmaps of descending aorta. Luminal (L) side to the top. Scale bars equal 50 µm. (D) Large area and (E) high-resolution Raman scans of WT and *Eln$^{KO}$* at postnatal day 1 and TCA false-color intensity distribution images for elastic fibers (green), collagens (red), ECM (cyan) and nuclei (blue). Scale bars equal 40 µm for large area scans. Scale bars equal 25 µm for high resolution scans. (See also Supplemental Table S1)

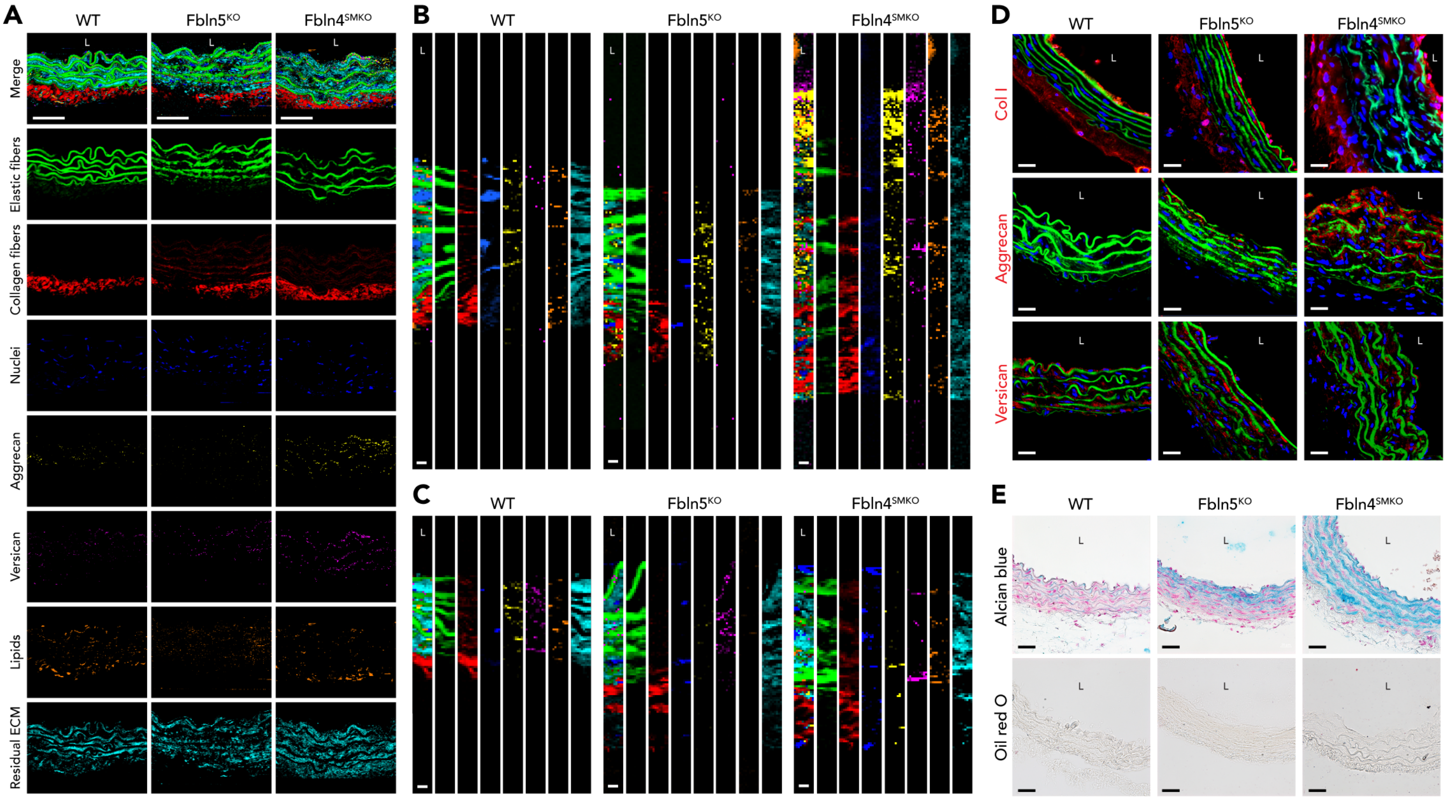


**Supplemental Figure S2. Representative large area and high-resolution Raman images and immunofluorescence (IF) staining and histochemistry of WT, *Fbln5$^{KO}$*, and *Fbln4$^{SMKO}$* descending aortas. Related to Figure 1.** (A) TCA false-color intensity distribution heatmaps for elastic fibers (green), collagen fibers (red), nuclei (blue), aggrecan (yellow), versican (pink), lipids (orange) and residual ECM (cyan) in adult WT, *Fbln5$^{KO}$*, and *Fbln4$^{SMKO}$* descending aortas. Scale bars equal 50 µm. (B-C) Raman images of high-resolution scans in (B) ascending and (C) descending aortic tissues for elastic fibers (green), collagen fibers (red), nuclei (blue), aggrecan (yellow), versican (pink), lipids (orange) and residual ECM (cyan). Scale bars equal 5 µm. (D) IF staining for collagen type Ⅰ, aggrecan, and versican (red), elastin autofluorescence (green) and nuclei (blue). Scale bars equal 20 µm. (E) Routine histochemical staining for Alcian blue (glycosaminoglycans) and Oil red O (lipid). Scale bars equal 20 µm.

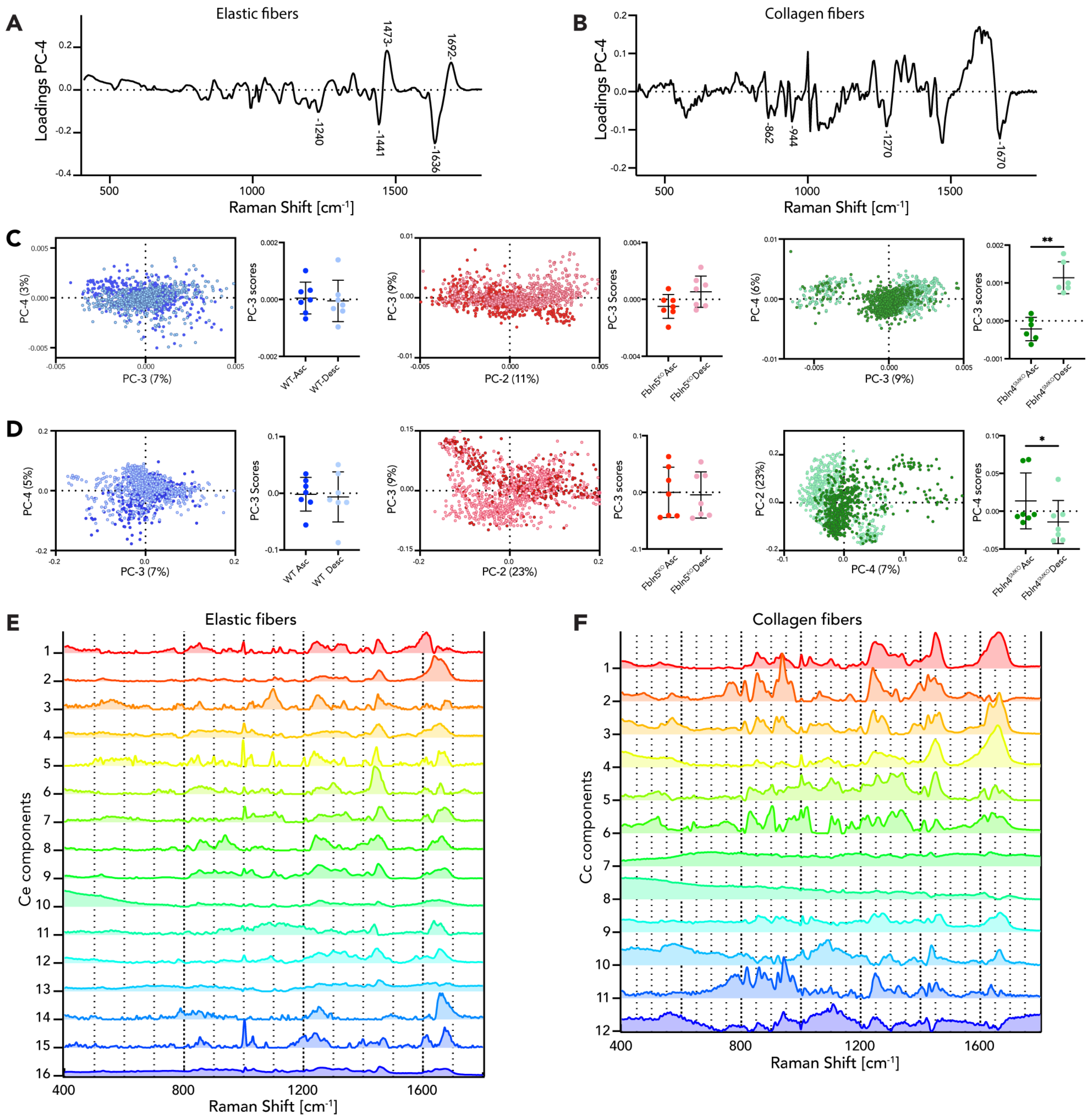


**Supplemental Figure S3. Murine MVA by PCA and MCR. Related to Figures 2, 3, and 4.** (A-B) PC loadings plots of (A) elastic fibers and (B) collagen fibers in murine ascending aortas shown in Figure 2. (C-D) PCA score plots of murine (C) elastic fibers and (D) collagen fibers comparing ascending (Asc) and descending (Desc) aortas in WT, *Fbln5*$^{KO}$, and *Fbln4*$^{SMKO}$. Both, elastic fibers and collagen fibers showed the separation between ascending and descending aortas in *Fbln4*$^{SMKO}$. Statistical analysis was performed by paired t-test (elastic fibers in WT, *Fbln5*$^{KO}$, and *Fbln4*$^{SMKO}$, and collagen fibers in WT and *Fbln5*$^{KO}$) and Wilcoxon test (collagen fibers in *Fbln4*$^{SMKO}$). *$p<0.05$, **$p<0.01$. N ≥ 6 per genotype. (E-F) MCR decomposed components of (E) elastic fibers (Ce1-Ce16 components) and (F) collagen fibers (Cc1-Cc12 components) of WT, *Fbln5*$^{KO}$, and *Fbln4*$^{SMKO}$. (See also Supplemental Table S2)

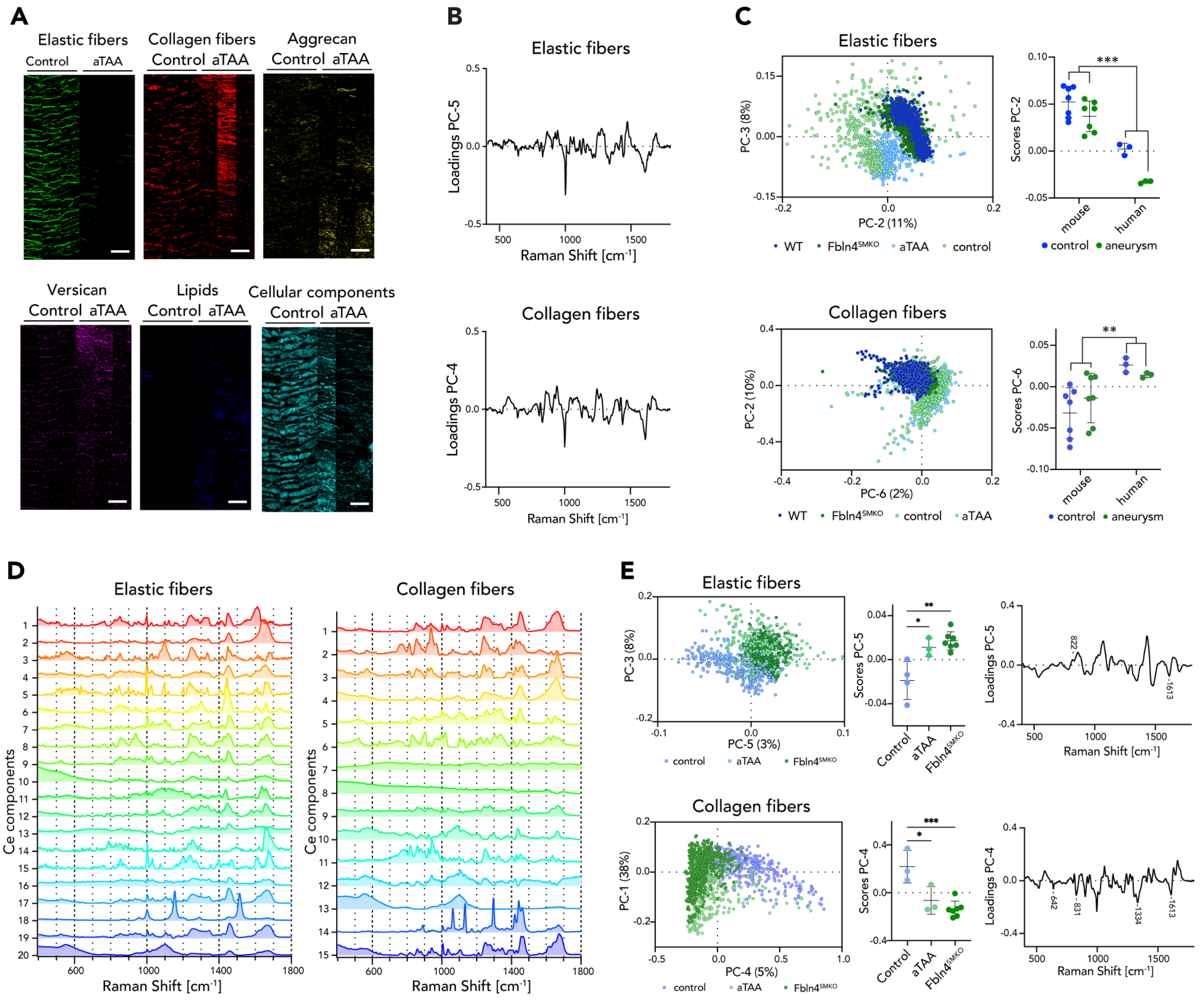


**Supplemental Figure 4. Human and murine MVA by TCA, PCA, and MCR for control and aTAA aortas. Related to Figures 5 and 6.** (A) TCA Raman imaging of human control aortas and aTAA obtained by murine reference spectra. Scale bars equal 50 µm. (B) Corresponding loadings plots to PCAs of human data (Figures 5B and 6B, respectively) performed in elastic fibers and collagen fibers. (C) PCA of murine and human tissues reveal species-specific differences in elastic fibers and collagens. PC-3 vs PC-2 scores plots of murine and human aortic elastic fibers. Statistical analysis of mean score values. PC-2 vs PC-6 scores plots of murine and human aortic collagen fibers. Statistical analysis of mean score values. N ≥ 6 (murine) and n ≥ 3 (human), data represent mean score values ± SD, two-way ANOVA, **p<0.01, ***p<0.001. (D) Human MCR decomposed spectra from elastic fibers (Ce1-Ce20) and collagen fibers (Cc1-Cc15). (E) PCA identified clustering of principal components of human and murine aneurysm structures from human control. (upper panel) Elastic fiber PCA of control human, aTAA, and *Fbln4$^{SMKO}$* for PC-3 vs PC-5 scores plots, statistical analysis of PC-5 (mean score values ± SD), and loadings plots of PC-5. (lower panel) Collagen fiber PCA comparing control human, aTAA, and *Fbln4$^{SMKO}$* scores plots, statistical analysis of PC-4 (mean score values ± SD), and loadings plots of PC-4. N ≥3, one-way ANOVA, *; p<0.05, **p<0.01, ***p<0.001. (See also Supplemental Table S2)
